# Heterologous versus homologous COVID-19 booster vaccination in previous recipients of two doses of CoronaVac COVID-19 vaccine in Brazil (RHH-001): a phase 4, non-inferiority, single blind, randomised study

**DOI:** 10.1016/S0140-6736(22)00094-0

**Published:** 2022-02-05

**Authors:** Sue Ann Costa Clemens, Lily Weckx, Ralf Clemens, Ana Verena Almeida Mendes, Alessandra Ramos Souza, Mariana B V Silveira, Suzete Nascimento Farias da Guarda, Maristela Miyamoto de Nobrega, Maria Isabel de Moraes Pinto, Isabela G S Gonzalez, Natalia Salvador, Marilia Miranda Franco, Renata Navis de Avila Mendonça, Isabelle Silva Queiroz Oliveira, Bruno Solano de Freitas Souza, Mayara Fraga, Parvinder Aley, Sagida Bibi, Liberty Cantrell, Wanwisa Dejnirattisai, Xinxue Liu, Juthathip Mongkolsapaya, Piyada Supasa, Gavin R Screaton, Teresa Lambe, Merryn Voysey, Andrew J Pollard, RHH-001 study team, Mustapha Bittaye, Danielle Woods, Sophie Davies, Holly Smith, Marta Ulaszewska, Helen Sanders, Reece Mabette, Sophie Vernon, Zara Valliji, Gracie Mead, Chitra Tejpal, Juyeon Park, Amy Beveridge, Ahmed Eldawi, Sally Felle, Mayara Fraga, Thaiane Muniz Martins, Claudia Loureiro Martins Medrado, Laiana Januse de Arruda Cordeiro Matos

**Affiliations:** a Oxford Vaccine Group, Department of Paediatrics, University of Oxford, Oxford, UK; b Institute of Global Health, University of Siena, Siena, Italy; c Department of Pediatrics, Universidade Federal de São Paulo, São Paulo, Brazil; d International Vaccine Institute, Seoul, Korea; e Escola Bahiana de Medicina e Saúde Pública, Salvador, Brazil; f General Medicine, Hospital São Rafael, Salvador, Brazil; g Instituto D'Or de Pesquisa e Ensino, Salvador, Brazil; h Universidade Federal de São Paulo, São Paulo, Brazil; i Department of Pediatrics, Universidade Federal da Bahia, Salvador, Brazil; j Gonçalo Moniz Institute, Fiocruz, Salvador, Brazil; k Wellcome Centre for Human Genetics, Nuffield Department of Medicine, University of Oxford, Oxford, UK; l Chinese Academy of Medical Science Oxford Institute, University of Oxford, Oxford, UK; m NIHR Oxford Biomedical Research Centre, Oxford, UK

## Abstract

**Introduction:**

The inactivated whole-virion SARS-CoV-2 vaccine (CoronaVac, Sinovac) has been widely used in a two-dose schedule. We assessed whether a third dose of the homologous or a different vaccine could boost immune responses.

**Methods:**

RHH-001 is a phase 4, participant masked, two centre, safety and immunogenicity study of Brazilian adults (18 years and older) in São Paulo or Salvador who had received two doses of CoronaVac 6 months previously. The third heterologous dose was of either a recombinant adenoviral vectored vaccine (Ad26.COV2-S, Janssen), an mRNA vaccine (BNT162b2, Pfizer–BioNTech), or a recombinant adenoviral-vectored ChAdOx1 nCoV-19 vaccine (AZD1222, AstraZeneca), compared with a third homologous dose of CoronaVac. Participants were randomly assigned (5:6:5:5) by a RedCAP computer randomisation system stratified by site, age group (18–60 years or 61 years and over), and day of randomisation, with a block size of 42. The primary outcome was non-inferiority of anti-spike IgG antibodies 28 days after the booster dose in the heterologous boost groups compared with homologous regimen, using a non-inferiority margin for the geometric mean ratio (heterologous *vs* homologous) of 0·67. Secondary outcomes included neutralising antibody titres at day 28, local and systemic reactogenicity profiles, adverse events, and serious adverse events. This study was registered with Registro Brasileiro de Ensaios Clínicos, number RBR–9nn3scw.

**Findings:**

Between Aug 16, and Sept 1, 2021, 1240 participants were randomly assigned to one of the four groups, of whom 1239 were vaccinated and 1205 were eligible for inclusion in the primary analysis. Antibody concentrations were low before administration of a booster dose with detectable neutralising antibodies of 20·4% (95% CI 12·8–30·1) in adults aged 18–60 years and 8·9% (4·2–16·2) in adults 61 years or older. From baseline to day 28 after the booster vaccine, all groups had a substantial rise in IgG antibody concentrations: the geometric fold-rise was 77 (95% CI 67–88) for Ad26.COV2-S, 152 (134–173) for BNT162b2, 90 (77–104) for ChAdOx1 nCoV-19, and 12 (11–14) for CoronaVac. All heterologous regimens had anti-spike IgG responses at day 28 that were superior to homologous booster responses: geometric mean ratios (heterologous *vs* homologous) were 6·7 (95% CI 5·8–7·7) for Ad26.COV2-S, 13·4 (11·6–15·3) for BNT162b2, and 7·0 (6·1–8·1) for ChAdOx1 nCoV-19. All heterologous boost regimens induced high concentrations of pseudovirus neutralising antibodies. At day 28, all groups except for the homologous boost in the older adults reached 100% seropositivity: geometric mean ratios (heterologous vs homologous) were 8·7 (95% CI 5·9–12·9) for Ad26.COV2-S vaccine, 21·5 (14·5–31·9) for BNT162b2, and 10·6 (7·2–15·6) for ChAdOx1 nCoV-19. Live virus neutralising antibodies were also boosted against delta (B.1.617.2) and omicron variants (B.1.1.529). There were five serious adverse events. Three of which were considered possibly related to the vaccine received: one in the BNT162b2 group and two in the Ad26.COV2-S group. All participants recovered and were discharged home.

**Interpretation:**

Antibody concentrations were low at 6 months after previous immunisation with two doses of CoronaVac. However, all four vaccines administered as a third dose induced a significant increase in binding and neutralising antibodies, which could improve protection against infection. Heterologous boosting resulted in more robust immune responses than homologous boosting and might enhance protection.

**Funding:**

Ministry of Health, Brazil.

**Research in context**

**Evidence before this study**

By Jan 17, 2022, 9·7 billion doses of COVID-19 vaccines had been deployed worldwide to reduce severe disease and death caused by the SARS-CoV-2. The most widely used vaccines were mRNA, viral vector, and inactivated vaccines, with widespread two-dose priming undertaken in low-income and middle-income countries with the inactivated vaccines from Sinovac and Sinopharm. As a result of waning immunity after two doses of COVID-19 vaccines and some evidence of reduced effectiveness, many countries are now considering offering third or booster doses. We searched PubMed for studies in English from Jan 1 to Dec 31, 2021 on booster doses of vaccines for individuals who had received two priming doses of the inactivated vaccine, CoronaVac. We found that heterologous boosting of CoronaVac with recombinant adenovirus type-5 COVID-19 vaccine produced greater neutralising antibody titres than did homologous boosting in a randomised trial in China. Similar findings are included in a preprint from Thailand comparing heterologous boosting with ChAdOx1 nCoV-19 (AstraZeneca), BNT162b2 (Pfizer–BioNTech), or BBIBP-CorV (Sinopharm), 3–4 months after CoronaVac.

**Added value of this study**

We report a comprehensive analysis of the immunogenicity and safety of homologous and heterologous boosting of the inactivated vaccine CoronaVac. We show that there are low concentrations of antibody present at 6 months after two doses of CoronaVac and largely undetectable neutralising antibodies. A third dose of CoronaVac boosts these responses and boosts are stronger with two different viral vector vaccines tested; the highest antibody concentrations are observed after an mRNA boost. We also show that heterologous boosting increases live virus neutralisation titres against both delta and omicron variants.

**Implications of all the available evidence**

Heterologous boosting of the inactivated vaccine, CoronaVac, results in more robust immune responses than homologous boosting and could enhance protection.

## Introduction

The inactivated whole-virion SARS-CoV-2 vaccine (CoronaVac; Sinovac Life Sciences, China and Instituto Butantan, Brazil) has been widely used in large-scale vaccination programmes in many countries.

In phase 3 randomised trials, two doses of CoronaVac showed varying levels of short-term efficacy against symptomatic COVID-19 (<6 months since vaccination), with efficacy and effectiveness estimates of 83·5% in Turkey,^1^ 50·7% in Brazil,^2^ and 65·9% in Chile.^3^ Efficacy against COVID-19 hospitalisation was higher with 83·7% (95% CI 58·0–93·7) efficacy in Brazil^2^and 87·5% (86·7 to 88·2) in Chile.^3^ In real-world use, a test-negative case control study in Brazil showed 46·8% (38·7–53·8) effectiveness against symptomatic infection and 55·5% (46·5–62·9) effectiveness against hospital admission during spread of the gamma (P.1) variant.^4^

Waning of immune responses has been observed after immunisation with COVID-19 vaccines, with reduced protection against infection and some loss of protection against hospitalisation and death, particularly among older adults. A third dose of CoronaVac (homologous boost) has been shown to be immunogenic.^5^, ^6^ However, boosting with a heterologous vaccine might provide greater immunity and protection against variants of concern. Heterologous boosting of CoronaVac with recombinant adenovirus type-5 COVID-19 vaccine produced greater neutralising antibody titres than did homologous boosting in a randomised trial in China.^7^ Similar findings have been observed in Thailand in a preprint comparing heterologous boosting with ChAdOx1 nCoV-19 (AstraZeneca), BNT162b2 (Pfizer–BioNTech), or BBIBP-CorV (Sinopharm), 3–4 months after CoronaVac.^8^ In mouse models, heterologous boosting of CoronaVac with one of three different vaccines resulted in better outcomes than did homologous boosting.^9^, ^10^

In this study, we compared the safety and immunogenicity of a third heterologous booster dose of one of three different vaccines, with a homologous boost in adults in Brazil who previously received two doses of CoronaVac.

## Methods

### Study design and participants

In RHH-001, we conducted a phase 4, randomised, participant blind, safety and immunogenicity study of a third heterologous booster dose of either the recombinant adenoviral vectored ChAdOx1 nCoV-19 vaccine (AZD1222, AstraZeneca, in combination with Fiocruz), mRNA vaccine (BNT162b2, Pfizer/BioNTech), or recombinant adenoviral vectored vaccine (Ad26.COV2-S, Janssen), compared with a third homologous boost with inactivated whole virion COVID-19 vaccine CoronaVac. The two study sites were in Brazil (Hospital São Rafael, Salvador, and CRIE UNIFESP, São Paulo).

Participants were eligible if they were 18 years or older; had received their second doses of CoronaVac 182 days (plus or minus 30 days) before enrolment; female participants were not pregnant, puerperal, or nursing; and all participants had given written informed consent. Participant exclusion criteria were history of laboratory-confirmed COVID-19 (or with fever or acute disease within 3 days before randomisation); serious vaccine-related adverse reactions; known bleeding disorders, neurological disorders, or history of Guillain-Barré syndrome; people with autoimmune disease (excluding people with Hashimoto's thyroiditis, vitiligo, psoriasis, lupus discords, HIV positive, or on HIV treatment); people on immunosuppressive medications within 15 days of vaccine; receipt of other investigational products, other vaccines within 14 days of enrolment or plans to receive vaccine within 28 days of vaccination, monoclonals within 9 months of day 1 or planned during the study, intravenous immunoglobulin, or other blood products; and any condition that could interfere with the primary objectives or represent additional risk to participants. Ethical approval was given by the National Ethical Review Committee, Comissão Nacional de Ética em Pesquisa.

### Randomisation and masking

Participants were randomly assigned to receive one of four different booster vaccines of either heterologous dosing with ChAdOx1 nCoV-19, BNT162b2, or Ad26.COV2-S, or homologous dosing with CoronaVac in a 5:6:5:5 ratio. The computer randomisation was conducted using RedCAP, stratified by site, age group (18–60 years or 61 years and older), and day of randomisation, with a block size of 42. Participants were enrolled from both age groups in equal numbers. The randomisation ratio was chosen to minimise vaccine wastage as the vaccines were supplied in five, six, or ten dose vials; therefore, 42 participants could be enrolled and vaccinated in a block with no wastage (appendix p 1). Participants were masked to the vaccine that they had received until the second visit, 28 days after vaccination. Blood samples for immunogenicity were taken before vaccination and at day 28 after vaccination. Study staff were aware of vaccine allocations, but laboratory staff remained masked.

### Procedures

CoronaVac is an inactivated COVID-19 vaccine; a 0·5 mL dose contains 600 SU of inactivated SARS-CoV-2 virus. ChAdOx1 nCoV-19 is a recombinant chimpanzee adenovirus that encodes full length spike SARS-CoV-2 glycoprotein; a 0·5 mL dose contains 5 × 10^10^ viral particles. BNT162b2 is a mRNA vaccine incorporated into lipid nanoparticles; a 0·3 mL dose contains 30 μg of SARS-CoV-2 spike protein messenger RNA. Ad26.COV2-S is a recombinant adenovirus type 26 that encodes SARS-CoV-2 spike glycoprotein used as a dose of 0·5 mL containing 5 × 10^10^ viral particles. All vaccines were administered intramuscularly.

A validated multiplexed immunoassay (3-plex ECL based assay on the MSD platform, PPD Vaccines, Richmond, VA, USA) was used to measure anti-spike, receptor binding domain, and nucleocapsid responses. The upper limit of the assay was 2 million arbitrary units per mililitre (AU/mL) and the lower limit was 1 AU/mL.

Antibody neutralisation titres on a random subset of 200 participants were measured with a lentivirus-based pseudovirus particle expressing the D614 SARS-CoV-2 spike protein (Monogram Biosciences, South San Francisco, CA, USA). Results are presented as inhibitory concentration of serum achieving 50% neutralisation of virus (IC_50_). The lower limit of the assay was 40 IC_50_.

A random subset of 80 participants (20 per group, stratified by age) were tested for live virus neutralisation using delta (B.1.617.2) and omicron (B.1.1.529) variants of SARS-CoV-2 virus with results reported as a value of 50% focus reduction neutralisation test (FRNT_50_), which is the reciprocal dilution of serum that neutralises 50% of the input virus. The lower limit was 20 FRNT_50_. For all assays, values above the upper limit were analysed at the upper limit, and values below the lower limit were substituted with half the lower limit. Samples were collected and stored locally before shipping to the centralised laboratories for testing.

### Outcomes

The primary outcome was non-inferiority of anti-spike IgG antibodies 28 days after the booster dose in the heterologous boost groups compared with homologous regimen. Secondary outcomes included neutralising antibody titres at day 28, local and systemic reactogenicity profiles self-reported by diary cards, adverse events, and serious adverse events (appendix pp 12–79).

### Statistical analysis

Antibody data were log-transformed before the analysis. The study used a non-inferiority design with the main hypothesis being that the anti-spike IgG induced by heterologous vaccine schedules is non-inferior to antibodies induced by the homologous vaccine schedule, using a non-inferiority margin for the geometric mean ratios (GMRs; heterologous *vs* homologous) of 0·67. GMRs were calculated by taking the anti-log of the mean difference between groups. Confidence intervals for the GMR with lower bounds greater than 0·67 were considered evidence of non-inferiority. Superiority comparisons were done where non-inferiority was shown using an unadjusted linear model fitted to log-transformed values with vaccine group as a fixed effect. To test the difference between response in younger and older adults, a linear model was fitted to log-transformed antibody values, adjusting for baseline antibody concentrations and vaccine group. The interaction term for vaccine group by age group was also tested but was not significant and was not included in the final model.

The primary analysis population included people who were randomly assigned, received at least one dose of the study vaccines or comparator, and provided post-vaccination immunogenicity data (ie, the modified intention-to-treat population). Missing data were not imputed. Confidence intervals for percentages were computed using the Binomial Exact (Clopper-Pearson) method. All analyses were done using R, version 4.1.1

Assuming a standard deviation of 0·4 for anti-spike IgG 28 days after the booster dose, 90% power, and alpha of 0·0167 due to three comparisons of heterologous versus homologous schedules, the study required 124 evaluable people per age group and per study group. Allowing for 20% loss to follow-up or incomplete data and the required randomisation ratio, 1240 people were planned for enrolment.

This study was registered with Registro Brasileiro de Ensaios Clínicos, number RBR – 9nn3scw.

### Role of the funding source

The study was funded by the Ministry of Health, Brazil and sponsored by Instituto D'Or de Pesquisa e Ensino. The Oxford investigators were supported by the NIHR Oxford Biomedical Research Centre. The funders had no role in the study design, data collection, data analysis, data interpretation, writing of the report, or in the decision to submit the paper for publication.

## Results

**Figure 1:**
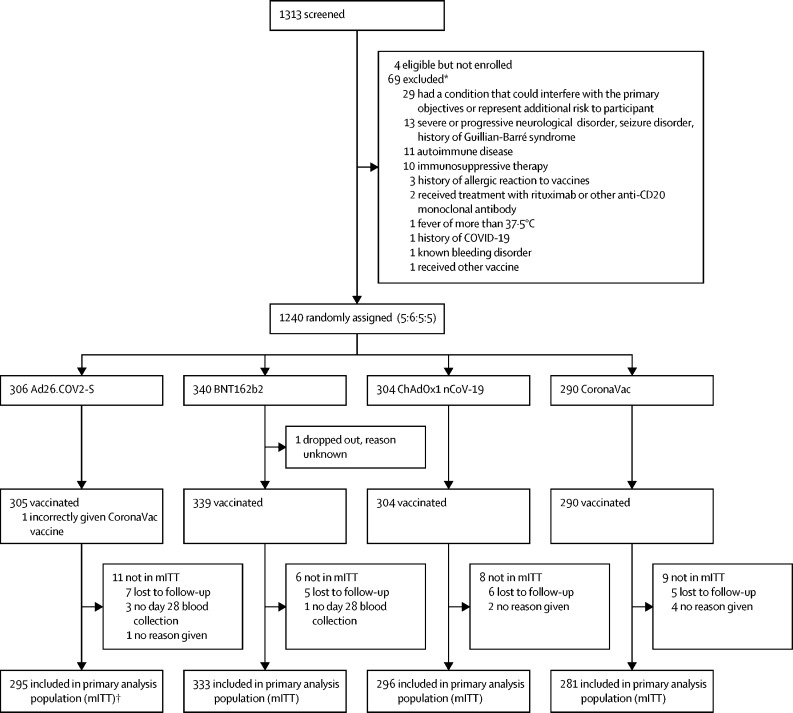
Trial profile mITT=modified intention to treat population. *It is possible to have more than one reason for exclusion per person; therefore, the number of people excluded is less than the sum of the reasons for exclusion. †The one person incorrectly given CoronaVac was included in the mITT.

Between Aug 16, and Sep 1, 2021, 1240 participants were randomly assigned in two age groups (18–60 years and 61 years or older), of whom 1239 were vaccinated. One participant was vaccinated with a vaccine to which they had not been randomly assigned (figure 1). 1205 (97%) returned for their day 28 visit and were eligible for inclusion in the primary analysis.

Participants included in the primary analysis ranged in age from 21 years to 98 years (median 60 years). The median time since receipt of the second dose of CoronaVac was 180 days (range 152–210). Of the 1205 participants, 729 (60·5%) were female and 814 (67·6%) were White. The most common pre-existing comorbidity was hypertension, present in 365 (30·3%) participants. Baseline characteristics were balanced across the four vaccine arms of the trial (table 1).

**Table 1:** Baseline characteristics of primary analysis population

	**Overall (n=1205)**	**Ad26.COV2-S (n=295)**	**BNT162b2 (n=333)**	**ChAdOx1 nCoV-19 (n=296)**	**CoronaVac (n=281)**
**Sex**					
Male	476 (39·5%)	114 (38·6%)	129 (38·7%)	117 (39·5%)	116 (41·3%)
Female	729 (60·5%)	181 (61·4%)	204 (61·3%)	179 (60·5%)	165 (58·7%)
**Age**					
18–60 years	616 (51·1%)	153 (51·9%)	165 (49·5%)	150 (50·7%)	148 (52·7%)
Over 61 years	589 (48·9%)	142 (48·1%)	168 (50·5%)	146 (49·3%)	133 (47·3%)
Median (range)	60 (21–98)	59 (22–98)	61 (21–95)	60 (21–96)	58 (21–95)
**Race**					
White	814 (67·6%)	203 (68·8%)	230 (69·1%)	200 (67·6%)	181 (64·4%)
Black	57 (4·7%)	14 (4·7%)	17 (5·1%)	13 (4·4%)	13 (4·6%)
Mixed	275 (22·8%)	65 (22·0%)	68 (20·4%)	70 (23·6%)	72 (25·6%)
Asian	57 (4·7%)	13 (4·4%)	17 (5·1%)	12 (4·1%)	15 (5·3%)
Not given	2 (0·2%)	0 (0%)	1 (0·3%)	1 (0·3%)	0 (0%)
**Medical history**					
Type 2 diabetes	127 (10·5%)	34 (11·5%)	21 (6·3%)	39 (13·2%)	33 (11·7%)
Heart failure	9 (0·7%)	3 (1·0%)	1 (0·3%)	2 (0·7%)	3 (1·1%)
COPD	9 (0·7%)	1 (0·3%)	2 (0·6%)	2 (0·7%)	4 (1·4%)
Hypertension	365 (30·3%)	84 (28·5%)	91 (27·3%)	99 (33·4%)	91 (32·4%)
Cancer	126 (10·5%)	27 (9·2%)	33 (9·9%)	38 (12·8%)	28 (10·0%)
Immunosuppressed	3 (0·2%)	1 (0·3%)	2 (0·6%)	0 (0%)	0 (0%)
Chronic kidney disease	7 (0·6%)	0 (0%)	1 (0·3%)	3 (1%)	3 (1·1%)
Coronary artery disease	61 (5·1%)	7 (2·4%)	18 (5·4%)	17 (5·7%)	19 (6·8%)
Cardiomyopathy	7 (0·6%)	2 (0·7%)	3 (0·9%)	1 (0·3%)	1 (0·4%)
Sickle cell anaemia	1 (0·1%)	0 (0%)	1 (0·3%)	0 (0%)	0 (0%)
Obesity	80 (6·6%)	24 (8·1%)	21 (6·3%)	20 (6·8%)	15 (5·3%)
HIV	2 (0·2%)	0 (0%)	2 (0·6%)	0 (0%)	0 (0%)
**Time since second vaccine, days**					
Mean (SD)	178·4 (9·9)	178·7 (9·6)	178·6 (10·1)	178·9 (9·7)	177·4 (10·3)

COPD=Chronic obstructive pulmonary disease.

**Figure 2:**
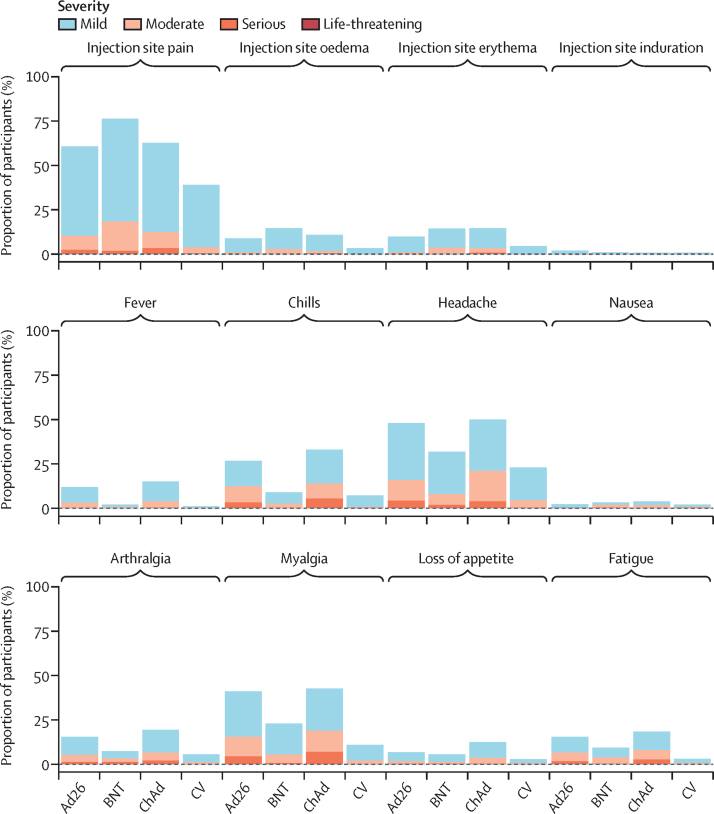
Local and systemic solicited adverse reactions in the first 7 days after vaccination (safety population) Ad26=Ad.26.COV2-S (n=305). BNT=BNT162b2 (n=339). ChAd=ChAdOx1 nCoV-19 (n=304). CV=CoronaVac (n=291).

The most common solicited local vaccine reaction in the first 7 days was injection site pain by 183 (60%) of 305 for Ad26.COV2-S, 256 (76%) 339 for BNT162b2, 192 (63%) of 304 for ChAdOx1 nCoV-19, and 114 (39%) of 291 for CoronaVac. Headaches were common for Ad26.COV2-S (137 [45%] of 305) and ChAdOx1 nCoV-19 recipients (148 [49%] of 304), compared with BNT162b2 (102 [30%] of 339) and CoronaVac (58 [20%] of 291). Myalgia was also commonly reported in 121 (40%) of 305 for Ad26.COV2-S group, in 77 (23%) of 339 for BNT162b2 group, 130 (43%) of 304 for ChAdOx1 nCoV-19, and in 30 (10%) of 291 recipients of CoronaVac. Fever and chills were common for Ad26.COV2-S (35 [11%] and 79 [26%] of 305) and ChAdOx1 nCoV-19 (44 [14%] and 99 [33%] of 304), but not for recipients of BNT162b2 (seven [2%] and 29 [9%] of 339) or CoronaVac (two [1%] and 21 [7%] of 291; figure 2).

There were five serious adverse events recorded. Three serious adverse events were considered possibly related to the vaccine received: in the BNT162b2 group, a woman of 83 years had a pulmonary embolism and deep vein thrombosis 2 days after vaccination; in the Ad26.COV2-S group, a woman of 52 years had a subconjunctival haemorrhage 2 days after vaccination, and a man of 71 years had a pulmonary embolism 28 days after vaccination. Unrelated serious adverse events included one case of bullous erysipelas (ChAdOx1 nCoV-19), and one case of coronary arterial disease requiring stent insertion (Ad26.COV2-S). All participants recovered and were discharged home. There were no COVID-19 cases identified during the study.

**Figure 3:**
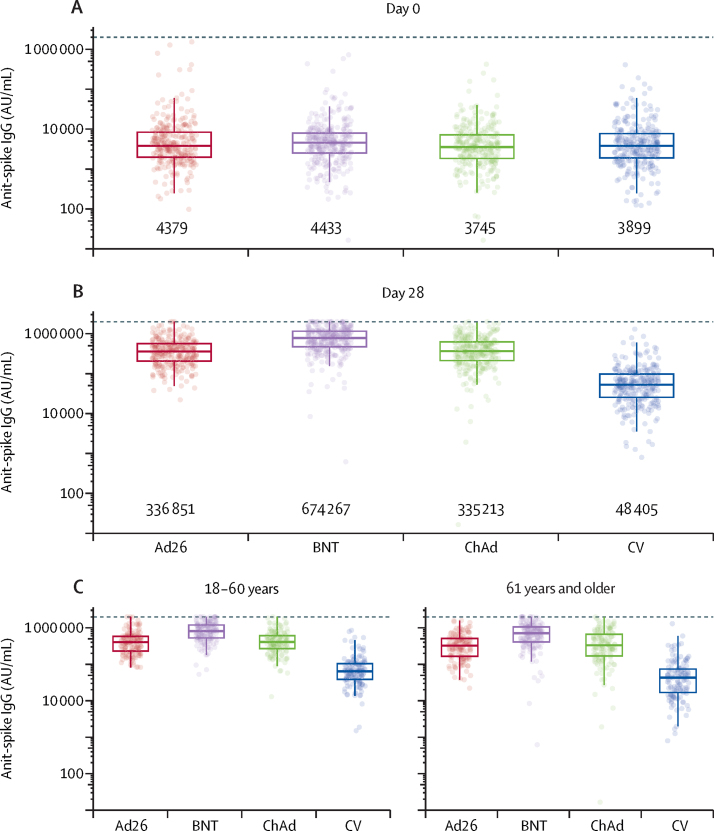
Anti-spike IgG by multiplex immunoassay by study day and age (A) Day 0, (B) day 28, and (C) day 28 responses by age group and booster vaccine allocation. Dotted line shows upper limit of the assay. The midlines of the boxes show medians and the outer bounds of the boxes show IQRs. Error bars extend to the last data point within 1·5 × the IQR above or below the 75th or 25th percentile. Geometric means shown below each group. See table 2 and appendix (p 3) summary statistics and comparisons. Ad26=Ad.26.COV2-S. AU/mL=arbitrary units per millilitre (conversion factor to convert AU/mL units to BAU/mL units using WHO Reference Standard is 0·00645 [95% CI 0·00594–0·00701]). BNT=BNT162b2. ChAd=ChAdOx1 nCoV-19. CV=CoronaVac.

At baseline there were no significant differences in anti-spike IgG across the four randomised groups (p=0·26). At day 28 after the booster vaccine all groups had a substantial rise in antibody concentrations (appendix p 3). The geometric fold-rise from baseline to day 28 was 77 (67–88) for Ad26.COV2-S, 152 (134–173) for BNT162b2, 90 (95% CI 77–104) for ChAdOx1 nCoV-19, and 12 (11–14) for CoronaVac (figure 3; appendix p 3).

All heterologous regimens were non-inferior to CoronaVac. Superiority comparisons were conducted and all heterologous regimens had anti-spike IgG at day 28 that was superior to that induced by the homologous boost (all p<0·0001, table 2). GMRs were 6·7 (95% CI 5·8–7·7) for Ad26.COV2-S, 13·4 (11·6–15·3) for BNT162b2, and 7·0 (6·1–8·1) for ChAdOx1 nCoV-19 (table 2, figure 3). Similar responses were seen with anti-receptor binding domain IgG (appendix p 7–8) but not with anti-nucleocapsid IgG, which was raised in participants receiving the CoronaVac boost containing the inactivated whole virus (appendix p 5–6). Responses in older adults were 19% lower than in younger adults at day 28, across all vaccines when tested in a linear model adjusted for vaccine group and baseline antibody levels (GMR 0·81 [95% CI 0·73–0·89] in 61 years and older *vs* 18–60 years, adjusted for vaccine group and baseline anti-spike IgG). In the older age group, the geometric fold-rise was 78·8 (95% CI 65·1–95·2) for Ad26.COV2-S, 165·4 (138·1–198·1) for BNT162b2, 91·5 (72·6–115·2) for ChadOx1 nCoV-19, and 12·5 (10·3–15·2) for CoronaVac.

**Table 2:** Comparisons of heterologous versus homologous regimens

		**Ad26.COV2-S**	**BNT162b2**	**ChAdOx1 nCoV-19**	**CoronaVac**	p value*
**Anti-spike IgG by multiplex immunoassay**						
All participants						
Number of participants		294	333	296	281	··
Geometric mean ratio		6·7 (5·8–7·7)†	13·4 (11·6–15·3)†	7·0 (6·1–8·1)†	ref	<0·0001
18–60 years						
Number of participants		152	165	150	148	··
Geometric mean ratio		6·1 (5·1–7·2)	12·1 (10·3–14·2)	6·4 (5·5–7·6)	ref	··
61 years and over						
Number of participants		142	168	146	133	··
Geometric mean ratio		7·3 (5·8–9·2)	15·0 (12·0–18·6)	7·6 (6·1–9·5)	ref	··
**Pseudovirus neutralisation titres**						
All participants						
Number of participants		47	49	52	46	··
Geometric mean ratio		8·7 (5·9–12·9)	21·5 (14·5–31·9)	10·6 (7·2–15·6)	ref	<0·0001
18–60 years						
Number of participants		22	23	26	22	··
Geometric mean ratio		7·2 (4·5–11·4)	15·6 (9·8–24·7)	8·2 (5·2–12·9)	ref	··
61 years and over						
Number of participants		25	26	26	24	··
Geometric mean ratio		10·5 (5·6–19·5)	30·7 (16·5–57·1)	14·2 (7·6–26·5)	ref	··

Data are the geometric mean ratio of heterologous versus homologous (95% CI), unless otherwise specified.

* p value from ANOVA model comparing log-geometric means across all four groups.

† p value <0·0001, values from superiority comparisons comparing heterologous schedules to homologous schedules.

**Figure 4:**
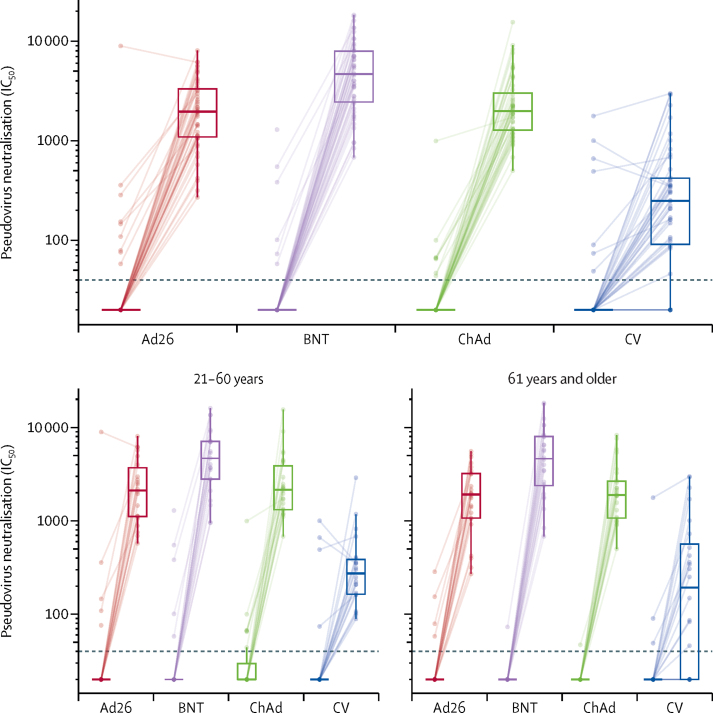
Pseudovirus neutralisation titres before and 28 days after boost vaccination by vaccine allocation and age group Lines connect values from the same participant. Dotted line shows lower limit of the assay. Values below the limit were substituted with a titre of 20. Participants with antibody titres above the lower limit are considered seropositive. The midlines of the boxes show medians and the outer bounds of the boxes show IQRs. Error bars extend to the last data point within 1·5 × the IQR above or below the 75th or 25th percentile. See table 2 and appendix (pp 7–8) for summary statistics. Ad26=Ad.26.COV2-S. BNT=BNT162b2. ChAd=ChAdOx1 nCoV-19. CV=CoronaVac. IC_50_=inhibitory concentration of serum achieving 50% neutralisation of virus (appendix pp 9–10).

Pseudovirus neutralisation titres were available on a random subset of 200 participants. 6 months after the second dose of CoronaVac and before the booster, 28 (14%) of the 194 participants (95% CI 9·8–20·2) had detectable neutralising antibodies on this assay. This value was lower in older adults (nine [9%] of 101, 95% CI 4·2–16·2) than in adults aged 18–60 years (19 [20%] of 93, 12·8–30·1; p=0·022). All participants in the three heterologous booster groups had neutralisation titres that were above the lower limit of detection 28 days after vaccination compared with 38 (83%) of 46 responders (95% CI 68·6–92·2) in the homologous CoronaVac arm. All heterologous regimens were superior to the homologous boost regimen (all p <0·0001), with GMRs of 8·7 (5·9–12·9) for Ad26.COV2-S, 21·5 (14·5–31·9) for BNT162b2, and 10·6 (7·2–15·6) for ChAdOx1 nCoV-19 (figure 4, table 2; appendix p 9–10).

**Figure 5:**
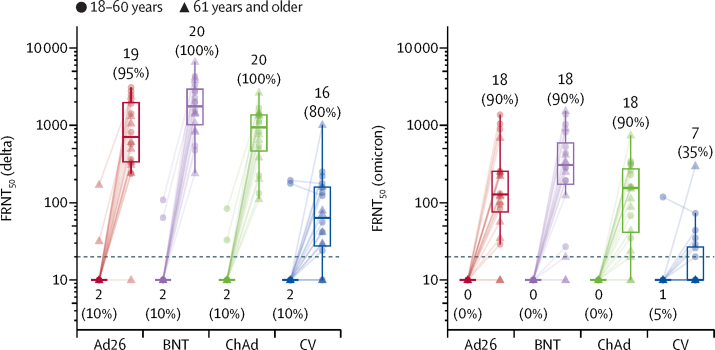
Live virus neutralisation titres against delta and omicron variant strains, before and 28 days after boost vaccination by booster vaccine groups In each group, ten samples were selected from each age group (18–60 years, 61 years and older). Lines connect values from the same participant. Dotted line shows lower limit of the assay. Values below the limit were substituted with a titre of 10. Participants with antibody titres above the lower limit are considered seropositive and are shown as percentages. The midlines of the boxes show medians and the outer bounds of the boxes show IQRs. Error bars extend to the last data point within 1·5 × the IQR above or below the 75th or 25th percentile. See appendix (p 14) for summary statistics. Ad26=Ad.26.COV2-S (n=20). BNT=BNT162b2 (n=20). ChAd=ChAdOx1 nCoV-19 (n=20). CV=CoronaVac (n=20). FRNT_50_=Focus reduction neutralisation test—the reciprocal dilution of serum that neutralises 50% of the input virus (appendix p 11).

Neutralising antibody titres measured by a live virus assay were above the lower limit of detection in 75 (94%) of 80 participants tested at day 28 for the delta variant and in 61 (76%) of 80 participants for the omicron variant (figure 5). The geometric mean titres for the four booster vaccines differed significantly at day 28 for both omicron and delta (both p<0·0001), but the ratio of omicron to delta did not differ between groups (p=0·11; appendix p 14).

## Discussion

In this study, we have shown that a third dose booster of the four vaccines tested provides a substantial increase in antibody responses after two doses of CoronaVac, when administered about 6 months after the second dose.

Very low neutralising antibody concentrations were detected at 6 months after two doses of the inactivated vaccine, CoronaVac, but both homologous and heterologous COVID-19 booster vaccinations were safe and strongly enhanced the humoral immune responses. The magnitude of the immune boost was greater with the adenoviral vectored vaccines (Ad26.COV2-S and ChAdOx1 nCoV-19) and mRNA vaccine (BNT162b2) than with the homologous regimen, with the highest responses reported after an mRNA boost, similar to recent findings following boosting with these vaccines after two priming doses of either mRNA or viral vector vaccines.^11^ In older adults, the difference in neutralising antibody titres was 8–22-fold higher for a heterologous boost than for a homologous boost with CoronaVac. In a preprint by Pan and colleagues,^6^ a third dose of CoronaVac given 6 months after the second dose resulted in an approximately 20-fold increase in neutralising antibody titres from a low baseline, higher than the 7-fold increase reported here for pseudoneutralising titres, or the 12-fold increase seen for anti-spike IgG. Differences in study population and laboratory assays might account for this discrepancy in absolute booster response, but substantial booster responses were observed in both studies. We also found that the booster doses of viral vector and mRNA vaccines substantially increased neutralising capacity of serum samples for both delta and omicron variants (at least 90% seropositive after booster), but lower responses were seen after a CoronaVac boost with just 35% becoming seropositive against omicron. Similarly, one preprint shows a 1·4-fold increase in anti-omicron neutralising capacity after an mRNA boost following two doses of CoronaVac, when compared with the activity of sera after two doses of the mRNA vaccine.^12^

One theoretical advantage of inactivated vaccines is that they contain additional viral proteins, including nucleoprotein, which could potentially broaden protection beyond anti-spike protein responses, and reduce the escape of variants from vaccine immunity. We show a 21-fold increase in anti-N IgG concentrations after the homologous boost but it is not clear whether these antibodies can confer clinical protection. Despite the addition of these anti-N responses, neutralising capacity of these sera is lower than those after a viral vector or mRNA boost, even though the latter responses are limited to anti-spike immunity.

Correlates of protection analysis of trial data from the UK phase 3 ChAdOx1 nCoV-19 vaccine efficacy trial showed that a median anti-spike IgG level of 139 306 AU/mL, and a pseudovirus neutralising antibody titre of 982 IC_50_ (140 IU/mL using the WHO international standard 20/136) was associated with 90% vaccine efficacy.^13^ Using the same assays, geometric mean antibody concentrations for the adenoviral-vectored vaccines in this study were 2·4-fold higher than the 90% vaccine efficacy correlate, and the mRNA vaccine had a geometric mean 4·8-times higher than the 90% correlate, suggesting that antibody concentrations in these groups would be associated with very high protection against symptomatic infection with variants circulating before February, 2021. After the booster, the CoronaVac group had a geometric mean titre that corresponded to the 80% vaccine efficacy correlate, using the values from Feng and colleagues.^13^

Immune responses are not always higher with heterologous boosting, highlighting the importance of generating primary data as shown here. Homologous boosting with a second or third dose of BNT162b2 produced higher antibody responses than a heterologous boost with an adenoviral-vectored vaccine (ChAdOx1 nCoV-19 or Ad26.COV2-S), an adjuvanted protein vaccine (NVX-CoV2373, Novavax), or a heterologous mRNA vaccine (CVnCov, CureVac).^11^, ^14^

WHO has not recommended widespread use of booster doses of COVID-19 vaccines due to continuing inequity in the distribution of first doses of vaccines to many parts of the world.^15^ However, in their interim statement on Dec 16, 2021, WHO advises that where countries are considering heterologous schedules, vectored or mRNA vaccines can be considered as third doses in those who received inactivated vaccines for initial doses.^16^ Our study shows that either of the four vaccines tested will produce a strong immune boost as a third dose after two doses of CoronaVac; however, heterologous boosting produced a substantially better response in this study. This finding might be especially relevant for the older adult population. It is not yet clear how long immunity will persist after a third dose and follow up at 6 months in this study will provide a comparison of antibody waning across the four vaccines tested.

The lowest reactogenicity was reported after CoronaVac boosting and the greater degree of reactogenicity seen with heterologous boosting in our study reflects similar findings from other randomised trials such as the Com-COV study, which compared homologous and heterologous boosting with ChAdOx1 nCoV-19 and BNT162b2 and found greater reactogenicity with heterologous schedules.^17^ Similarly, the COV-BOOST study of third doses of seven different vaccines showed greater reactogenicity in some heterologous schedules: mRNA-1273 after two doses of ChAdOx1 nCoV-19 or two doses of BNT162b2; and ChAdOx1 nCoV-19 or Ad26.COV2-S after two doses of BNT162b2.^11^

There are some limitations to this study. The study was single-blind for participants until their day 28 visit to ensure recording of vaccine reactions was not influenced by knowledge of the product received, but study staff were aware of vaccine allocations. However, the main outcomes were laboratory measures of antibody values and laboratory staff remained masked. This study was done only in Brazil and so it is not known whether these findings will translate to other populations, although two geographically distinct sites were used in an ethnically diverse population. Although not all available vaccines could be tested, a range of platforms were assessed, including inactivated vaccines, viral vectors, and mRNA, representing the products most widely available in populations where inactivated vaccines have been deployed. We present antibody data only because peripheral blood mononuclear cells, for use in T cell assays, were not collected in this study, and so it is not possible to speculate on the relative merits of the different schedules in inducing cellular immunity. In a previous study, after two doses of ChAdOx1 nCoV-19 or two doses of BNT162b2, T cells responses were induced with heterologous boosting regimens, but an inactivated vaccine (Valneva) did not induce T cell responses when used to boost either mRNA or viral vector vaccines.^11^

In conclusion, this study shows that use of all four vaccines as a third dose is safe and provides a strong immune response that is more robust than when a heterologous vaccine is used.

## Data sharing

Data are provided as a CSV file in supplementary material.

## Declaration of interests

AJP is chair of the UK Department of Health and Social Care's Joint Committee on Vaccination and Immunisation, but does not participate in policy advice on coronavirus vaccines, and is a member of the WHO Strategic Advisory Group of Experts. AJP is an National Institute for Health Research Senior Investigator. TL is named as an inventor on a patent application covering ChAdOx1 nCoV-19. Oxford University has entered into a partnership with AstraZeneca for further development of ChAdOx1 nCoV-19. All other authors declare no competing interests.
