# Supplementary material for: Heterologous versus homologous COVID-19 booster vaccination in previous recipients of two doses of CoronaVac COVID-19 vaccine in Brazil (RHH-001): a phase 4, non-inferiority, single blind, randomised study
Source: Lancet. 2022 Feb 5;399(10324):521–9. doi: 10.1016/S0140-6736(22)00094-0 (PMC8782575; doi:10.1016/S0140-6736(22)00094-0)
Supplement: Supplementary appendix [file mmc1.pdf]

THE LANCET

Supplementary appendix

This appendix formed part of the original submission and has been peer reviewed. We post it as supplied by the authors.

Supplement to: Costa Clemens SA, Weckx L, Clemens R, et al. Heterologous versus homologous COVID-19 booster vaccination in previous recipients of two doses of CoronaVac COVID-19 vaccine in Brazil (RHH-001): a phase 4, non-inferiority, single blind, randomised study. *Lancet* 2022; published online Jan 21. https://doi.org/10.1016/S0140-6736(22)00094-0.

Supplementary Material

Contents

Supplementary Randomisation Information 2

Table S1 Anti-spike IgG by multiplex immunoassay (Primary analysis population) 3

Table S2 Anti-N IgG by multiplex immunoassay in primary analysis population 5

Table S3 Anti-RBD IgG by multiplex immunoassay in primary analysis population 7

Table S4 Pseudovirus neutralising antibody titres (IC50) 9

Table S5 Live virus neutralising antibody titres against Delta and Omicron variants in a random subset of 80 participants 11

RHH-001 Protocol version 3.0 (English) 12

Supplementary Randomisation Information

The vaccines had different shelf lives and number of doses per vial. Therefore, randomisation was performed in blocks of 42 doses period to minimise the potential for vaccine wastage. Randomisation used a 5:6:5:5 ratio, stratified by age group and day of randomisation. Each new day involved opening new vials of vaccine and therefore sites aimed for complete enrolment of full blocks in multiples of 42 each calendar day.

Randomisation lists were generated by a statistician who was not further involved in analysis of study data, and uploaded into RedCAP for use during the study. Study staff randomised the participants using the RedCAP CRF with full allocation concealment.

The table below summarizes information on vaccine doses and vials and the randomisation ratio.

Vaccine	Expiry Time after opening vial	Number of doses/vial	Number of vials per block of randomisation (# of doses)	Randomisation ratio
ChAdOx1 nCoV-19	48h	5	2 (10)	5
BNT162b2	6h	6	2 (12)	6
Ad26.COV-2	6h	5	2 (10)	5
CoronaVac	8h	10	1 (10)	5

Table S1 Anti-spike IgG by multiplex immunoassay (Primary analysis population)

Age	Day		Ad26.COV2-S	BNT162b2	ChAdOx1 nCoV-19	CoronaVac
All		N	295	333	296	281
	Day 1	Geometric Mean	4379	4433	3745	3899
		95% Confidence Interval	3760, 5099	3880, 5064	3252, 4313	3351, 4537
	Day 28	Geometric Mean	336851	674267	335213	48405
		95% Confidence Interval	308342, 367997	615633, 738487	295598, 380136	42491, 55142
	Day 28/Day 1	N	294	333	296	281
		GMFR	76.8	152.1	89.5	12.4
		95% Confidence Interval	66.6, 88.4	133.5, 173.3	77.2, 103.8	10.8, 14.2
Age Groups						
18-60		N	153	165	150	148
	Day 1	Geometric Mean	5061	5479	4495	5057
		95% Confidence Interval	4117, 6221	4500, 6671	3798, 5321	4221, 6060
	Day 28	Geometric Mean	380572	765118	394096	62503
		95% Confidence Interval	340998, 424738	692029, 845925	349738, 444080	53810, 72600
	Day 28/Day 1	N	152	165	150	148
		GMFR	75	139.6	87.7	12.4

Age	Day		Ad26.COV2-S	BNT162b2	ChAdOx1 nCoV-19	CoronaVac
		95% Confidence Interval	60.7, 92.5	115.6, 168.7	72.6, 105.9	10.2, 15
Over 60		N	142	168	146	133
	Day 1	Geometric Mean	3750	3600	3104	2919
		95% Confidence Interval	2995, 4696	3020, 4292	2476, 3891	2292, 3718
	Day 28	Geometric Mean	295348	595547	283866	36422
		95% Confidence Interval	257098, 339289	512754, 691710	227361, 354415	29499, 44969
	Day 28/Day 1	N	142	168	146	133
		GMFR	78.8	165.4	91.5	12.5
		95% Confidence Interval	65.1, 95.2	138.1, 198.1	72.6, 115.2	10.3, 15.2

Conversion Factor to convert AU/mL units to BAU/mL units using WHO Reference Standard (20/136) is 0.00645 (95% CI 0.00594, 0.00701).

Table S2 Anti-N IgG by multiplex immunoassay in primary analysis population

Age	Day		Ad26.COV2-S	BNT162b2	ChAdOx1 nCoV-19	CoronaVac
All		N	295	333	296	281
	Day 1	Geometric Mean	536.7	583.7	498.4	505.6
		95% Confidence Interval	440.6, 653.7	478.8, 711.6	410.8, 604.7	408, 626.5
	Day 28	Geometric Mean	522.6	567.1	469.1	10619.7
		95% Confidence Interval	423.9, 644.3	464.5, 692.3	385.9, 570.2	8524, 13230.8
	Day 28/Day 1	N	294	333	296	281
		GMFR	1	1	0.9	21
		95% Confidence Interval	0.9, 1	0.9, 1	0.9, 1	17.4, 25.3
18-60		N	153	165	150	148
	Day 1	Geometric Mean	740.6	1027	759.9	892.9
		95% Confidence Interval	577.1, 950.5	774.6, 1361.6	585.6, 986.2	682.1, 1168.9
	Day 28	Geometric Mean	681.7	979.5	718.8	21465.8
		95% Confidence Interval	528, 880.2	740.1, 1296.2	547.2, 944.2	17364.5, 26535.8
	Day 28/Day 1	N	152	165	150	148
		GMFR	0.9	1	0.9	24
		95% Confidence Interval	0.8, 1	0.9, 1	0.9, 1	18.5, 31.2
Over 60		N	142	168	146	133

Age	Day		Ad26.COV2-S	BNT162b2	ChAdOx1 nCoV-19	CoronaVac
	Day 1	Geometric Mean	380.2	335.1	323.1	268.5
		95% Confidence Interval	281.1, 514.2	259.9, 432.2	246.2, 424	197.1, 365.8
	Day 28	Geometric Mean	392.5	331.6	302.5	4853
		95% Confidence Interval	281.2, 547.7	255.1, 431	232.3, 394	3391.6, 6944.1
	Day 28/Day 1	N	142	168	146	133
		GMFR	1	1	0.9	18.1
		95% Confidence Interval	0.9, 1.2	0.9, 1.1	0.9, 1	13.8, 23.7

Table S3 Anti-RBD IgG by multiplex immunoassay in primary analysis population

Age	Day		Ad26.COV2-S	BNT162b2	ChAdOx1 nCoV-19	CoronaVac
All		N	295	333	296	281
	Day 1	Geometric Mean	4558.7	4780.1	3957.3	3897.7
		95% Confidence Interval	3868.5, 5372.1	4147.4, 5509.3	3408.7, 4594.2	3296, 4609.3
	Day 28	Geometric Mean	430521.6	827704.7	447510.2	59445.8
		95% Confidence Interval	390411.9, 474752.2	754136.9, 908449.3	394652, 507448.2	51861.4, 68139.4
	Day 28/Day 1	N	294	333	296	281
		GMFR	94.2	173.2	113.1	15.3
		95% Confidence Interval	80.8 109.9	150.5 199.3	96.4 132.6	13.2 17.6
18-60		N	153	165	150	148
	Day 1	Geometric Mean	5506.9	6002.4	5044.8	5426.6
		95% Confidence Interval	4451.2, 6813.1	4877.4, 7386.9	4225.2, 6023.4	4478.1, 6576
	Day 28	Geometric Mean	499357.2	937168.4	539116.9	80848.8
		95% Confidence Interval	448598.4, 555859.3	850063.4, 1033198.8	481381.6, 603776.8	70004, 93373.6
	Day 28/Day 1	N	152	165	150	148
		GMFR	90.4	156.1	106.9	14.9
		95% Confidence Interval	72.6 112.5	127.7 190.8	87.6 130.4	12.2 18.2
Over 60		N	142	168	146	133

Age	Day		Ad26.COV2-S	BNT162b2	ChAdOx1 nCoV-19	CoronaVac
	Day 1	Geometric Mean	3723.9	3822.2	3083.6	2697
		95% Confidence Interval	2898.4, 4784.6	3160.7, 4622.2	2433.1, 3908.1	2055.5, 3538.8
	Day 28	Geometric Mean	366934.6	732649.9	369578.9	42218.9
		95% Confidence Interval	311263.8, 432562.3	626469.6, 856826.7	295375.6, 462423.2	33612.9, 53028.2
	Day 28/Day 1	N	142	168	146	133
		GMFR	98.5	191.7	119.9	15.7
		95% Confidence Interval	79.1 122.7	157.3 233.5	93.2 154.2	12.7 19.3

Table S4 Pseudovirus neutralising antibody titres (IC50)

Age	Day		Ad26.COV2-S	BNT162b2	ChAdOx1 nCoV-19	CoronaVac	P Value
All		N	47	49	52	46	
	Day 1	Geometric Mean	30.5	24.7	25.2	30.1	0.7152
		95% Confidence Interval	21.9, 42.4	20.2, 30.1	20.9, 30.2	21.7, 41.8	
	Day 28	Geometric Mean	1843.6	4325.8	2136.9	211.1	<0.0001
		95% Confidence Interval	1445.7, 2351	3403.6, 5498	1753.9, 2603.6	136.8, 325.6	
	Day 28/Day 1	N	47	49	52	46	
		GMFR	60.5	175.5	84.9	7.0	
		95% Confidence Interval	42.5, 86.1	135.2, 227.8	64.1, 112.5	4.4, 11.1	
18-60		N	22	23	26	22	
	Day 1	Geometric Mean	35.6	29.5	30.6	34.4	
		95% Confidence Interval	18.6, 68.1	19.5, 44.6	21.5, 43.8	19.7, 60	
	Day 28	Geometric Mean	2071.5	4396.9	2327.6	288.2	
		95% Confidence Interval	1460.6, 2937.9	3106.2, 6224	1740.4, 3113	197.3, 420.9	
	Day 28/Day 1	N	22	23	26	22	
		GMFR	58.2	149	76	8.4	
		95% Confidence Interval	31.2, 108.7	97.3, 228.1	46.4, 124.4	4.5, 15.6	
Over 60		N	25	26	26	24	

Age	Day		Ad26.COV2-S	BNT162b2	ChAdOx1 nCoV-19	CoronaVac	P Value
	Day 1	Geometric Mean	26.6	21	20.7	26.6	
		95% Confidence Interval	19.8, 35.7	19, 23.3	19.3, 22.1	17.8, 40	
	Day 28	Geometric Mean	1663.9	4263.9	1961.9	158.7	
		95% Confidence Interval	1164.6, 2377.2	2992, 6076.6	1477.2, 2605.6	73.4, 342.7	
	Day 28/Day 1	N	25	26	26	24	
		GMFR	62.5	202.8	94.9	6	
		95% Confidence Interval	41, 95.3	145.3, 283.2	70, 128.8	2.9, 12.2	

IC50: Inhibitory concentration resulting in 50% neutralisation. P value from ANOVA model comparing log-geometric means across all groups. Separate tests not presented for age groups due to smaller numbers in the subgroups. Conversion factor to convert IC50 units to IU/mL units using WHO Reference Standard (20/136) is 0.1458.

Table S5 Live virus neutralising antibody titres against Delta and Omicron variants in a random subset of 80 participants

	Day	Ad26.COV2-S N=20	BNT162b2 N=20	ChAdOx1 nCoV-19 N=20	CoronaVac N=20	P value
Delta	Day 0	12 (9, 17)	12 (9, 17)	12 (9, 15)	13 (9, 20)	
	Day 28	677 (364, 1257)	1653 (1118, 2443)	710 (467, 1080)	61 (34, 111)	<0.0001
Omicron	Day 0	10 (10, 10)	10 (10, 10)	10 (10, 10)	11 (9, 15)	
	Day 28	138 (72, 264)	223 (108, 458)	102 (57, 182)	17 (11, 26)	<0.0001
Ratio Omicron/Delta	Day 28	0.20 (0.13, 0.33)	0.13 (0.08, 0.22)	0.14 (0.08, 0.25)	0.28 (0.19, 0.41)	0.1063

Data shown are geometric means and 95% confidence intervals. P value from ANOVA model comparing log-geometric means across all groups

CLINICAL TRIAL PROTOCOL

Title: A phase 4, randomized, controlled, single-blind study to assess the immunogenicity and safety of a third heterologous booster dose with recombinant covid-19 vaccine (AstraZeneca/Fiocruz), mRNA covid-19 vaccine (Pfizer/Wyeth) or recombinant covid-19 vaccine (Janssen) in previously vaccinated subjects against Covid-19 with two Sinovac/Butantan doses compared to a third dose of homologous booster dose of adsorbed inactivated covid-19 vaccine (Sinovac/Butantan) in adults.

Brief title: Immunogenicity and safety of heterologous booster vaccination with recombinant covid-19 vaccine (AstraZeneca/Fiocruz), mRNA covid-19 vaccine (Pfizer/Wyeth) or recombinant covid-19 vaccine and homologous inactivated covid-19 vaccine (Sinovac/Butantan).

Protocol Number:	RHH_001
Vaccines:	• Recombinant covid-19 vaccine (AstraZeneca/Fiocruz)<br>• mRNA covid-19 vaccine (Pfizer/Wyeth)<br>• Recombinant covid-19 vaccine (Janssen)<br>• Adsorbed inactivated covid-19 vaccine (Sinovac/Butantan)
Study phase	4
Sponsor Name:	Instituto D'Or de Pesquisa e Ensino (IDOR)
Funding:	Ministry of Health - Brazil
Version:	3.0
Protocol date	13/Dec/2021

This document is confidential. It contains information proprietary of IDOR. The use of this information without previous written authorization by IDOR is strictly forbidden. Information may be used solely for purposes of reviewing and conducting this study.

CONFIDENTIAL

MAIN STUDY CONTACTS

National Coordinating Investigator: Prof. Dr. Sue Ann Costa Clemens

Director and Professor of the Vaccine Group of the University of Oxford – United Kingdom

Director of Global Health Institute of University of Siena - Italy

Local Principal Investigator: Dr. Ana Verena Almeida Mendes

Hospital São Rafael - Rede D'Or - Salvador

Co-Investigators

Prof. Dr. Andrew Pollard

University of Oxford – United Kingdom

Dr. Ralf Clemens

International Vaccine Institute - Korea

Prof. Dr. Lily Weckx

UNIFESP – Brazil

Study sites:

Investigator in Charge:	Ana Verena Almeida Mendes
Study site:	Hospital São Rafael
	Av. São Rafael, 2152, São Marcos, Salvador, BA, Zip Code: 41253-190

Investigator in Charge:	Lily Weckx
Study site:	CRIE UNIFESP
	Av. Borges Lagoa, 770, São Paulo, SP, Zip Code: 04038-001

PROTOCOL SIGNATORIES

I read the full protocol and agree to carry out the study as per:

Prof. Dr. Sue Ann Costa Clemens<br>National Coordinator and Investigator:<br>Principal Co-Investigator	Date
Dr. Ana Verena Almeida Mendes<br>Local Principal Investigator	Date

TABLE OF CONTENTS

MAIN STUDY CONTACTS **2**

PROTOCOL SIGNATORIES **3**

TABLE OF CONTENTS **4**

LIST OF TABLES **7**

LIST OF FIGURES **7**

LIST OF ABBREVIATIONS **8**

1.0 PROTOCOL SYNOPSIS **9**
- **1.1 Study design** **16**
- **1.2 Schedule of activities** **17**

2.0 INTRODUCTION **19**
- **2.1 Study rationale** **20**
  - 2.1.1 Scientific rationale for the study design 20
- **2.2 Risk/benefit assessment** **22**
  - 2.2.1 Risk assessment 22
  - 2.2.2 Benefit Assessment 22
  - 2.2.3 Overall risk/benefit conclusion 23

3.0 OBJECTIVES, ENDPOINTS AND ESTIMATIONS **24**

4.0 STUDY DESIGN **26**
- **4.1 Overall design** **26**
- **4.2 End of study definition** **26**

5.0 STUDY POPULATION **27**
- **5.1 Inclusion criteria** **27**
- **5.2 Exclusion criteria** **27**
- **5.3 Previous and concomitant medications** **28**
- **5.4 Forbidden Drugs** **28**
- **5.5 Screening failures** **29**

6.0 VACCINES/COMPARATORS 30
6.1 Subject randomization and blinding 31
6.2 Study vaccine data recording 31

7.0 SUBJECT WITHDRAWAL FROM THE STUDY 33
7.1 Early withdrawal 33
7.2 Subject withdrawal 33
7.3 Loss of follow-up 33

8.0 STUDY ASSESSMENTS AND PROCEDURES 34
8.1 Informed Consent Form 34
8.2 Demographics 34
8.3 Physical Exam and Vital Signs 34
8.4 Pregnancy Test 35
8.5 Inclusion and exclusion criteria 35
8.6 Randomization 35
8.7 Vaccination 36
8.8 Immunogenicity assessments 36
8.8.1 Blood volumes and serological tests 37
8.8.2 Biological sample retention and destruction 37
8.9 Safety assessments 37
8.10 Schedule of activities 38
8.10.1 Pre-vaccination procedures (Day 1) 38
8.10.2 Vaccination and post-vaccination procedures (Day 1) 38
8.10.3 Last visit procedures (Day 28) 39

9.0 ADVERSE EVENTS 41
9.1 Definitions 41
9.1.1 Definition of an adverse event 41
9.1.2 Definition of a SAE 42
9.1.3 AEs and SAEs Recording 43
9.1.4 Intensity assessment 43
9.1.5 Causality assessment 43
9.1.6 AE outcome assessment 44
9.1.7 Time period and frequency to collect information on adverse and serious events 45
9.1.8 Methods to detect AEs and/or SAEs 45
9.1.9 Solicited and spontaneous adverse events 45
9.1.10 AEs and SAEs Follow-up 48
9.1.11 SAE regulatory report requirements 48
9.1.12 AESIs and SAEs Reports 49
9.1.13 Pregnancy 49
9.1.14 Adverse events of special interest 49

9.1.15 Adverse events with medical care 50
9.1.16 Assessment of Potential COVID-19 Cases 50
9.2 Overdose treatment 51

10.0 STATISTICAL CONSIDERATIONS 52
10.1 Statistical hypotheses 52
10.2 Sample Size Determination 52
10.3 Statistical Analysis 53
10.3.1 Demographics and baseline characteristics analysis 54
10.3.2 Immunogenicity analyses 54
10.3.1 Safety analyses 55
10.4 Analysis timepoint..55

11.0 REFERENCE..56

12.0 APPENDIXES 57

LIST OF TABLES

Table 1 Schedule of Activities 17
Table 2 Major risks and mitigation strategy 22
Table 3 Objectives, endpoints and estimations 24
Table 4 Subject Disposition 26
Table 5 Immunogenicity assessments 37
Table 6 Test plan 37
Table 7 Seriousness rating for solicited local reactions and systemic adverse events 47
Table 8 Analysis sets 53
Table 9 Immunogenicity assessments 54
Table 10 Safety analyses 55
Table 11 Study Administrative Structure 61
Table 12 List of adverse events of special interest for the study 64
Table 13 List of adverse events of special interest relevant to COVID-19 (guidance document of the safety platform for emergency vaccine [SPEAC]) 67

LIST OF FIGURES

Figure 1 Study Design 16

LIST OF ABBREVIATIONS

ACE2	angiotensin converting enzyme 2
AdV	adenovirus
EUA	emergency use authorization
GCP	Good Clinical Practice
SoA	schedule of activities
COVID-19	coronavirus disease 2019
CRO	Contract Research Organization
IEC	Independent ethics committee
AE	adverse event
MAAEMAAE	medically-attended adverse event
SAE	Serious adverse event
AESI	adverse event of special interest
eCRF	electronic case report form
EDC	electronic data capture
ELISA	enzyme-linked immunosorbent assay
IMEs	important medical events
FSH	follicle-stimulating hormone
GMFR	geometric mean fold rise
GMP	Good Manufacturing Practices
GMT	geometric mean of the titer
HIV	human immunodeficiency virus
IM.	intramuscular
ICH	International Conference on Harmonization
IRT	Interactive response technology
LLOQ	lower limit of quantification
PPS	Per Protocol Set
SARS-CoV-2	acute respiratory syndrome coronavirus 2
ICF	informed consent form
NT	Neutralization Titer
VNA	Virus Neutralization Assay

1.0 PROTOCOL SYNOPSIS

Sponsor	Protocol Number	Registration no.:	Date
IDOR	RHH_001	RBR – 9nn3scw	December 13,, 2021

Study Title: A phase 4, randomized, controlled, single-blind study to assess the immunogenicity and safety of a third heterologous booster dose with recombinant covid-19 vaccine (AstraZeneca/Fiocruz), mRNA covid-19 vaccine (Pfizer/Wyeth) or recombinant covid-19 vaccine (Janssen) in previously vaccinated subjects against Covid-19 with two Sinovac/Butantan doses compared to a third dose of homologous booster dose of adsorbed inactivated covid-19 vaccine (Sinovac/Butantan) in adults.

Publication (reference): Not applicable

Study Duration: 6 months	Clinical Phase: 4

Background and Rationale:

Coronavirus disease 2019 (COVID-19) is caused by novel coronavirus referred to as SARS-CoV-2, which was first identified in Wuhan, China, in December 2019, after a report of pneumonia of unknown causes. This is a highly transmissible virus and serious COVID-19 cases require hospitalization in intensive care units (ICU) requiring mechanical ventilation and have a high associated mortality. Globally, in July 6, 2021, there were 183,934,913 confirmed COVID-19 cases, including 3,985,022 deaths reported to the World Health Organization (WHO). In Brazil, there were 18,769,808 cases and 524,417 confirmed deaths. In fact, during 2021, cases continue to increase in several countries and phenomena of "second and third waves" of infection. In this context, it is worth pointing out that vaccination is one of the most effective strategies to eradicate infectious diseases. Vaccines authorized for use, and most vaccines under development, have SARS-CoV-2 spike (S) protein as the main antigenic virus target. In Brazil, ANVISA granted an authorization for recombinant covid-19 vaccine (AstraZeneca/Fiocruz) as a 2-dose homologous vaccination regimen, with a window of 28 to 84 days, and for mRNA covid-19 vaccine (Pfizer/Wyeth) in a 2-dose homologous vaccination regimen, with a window of 21-84 days. Moreover, emergency use authorization (EUA) was granted to adsorbed inactivated covid-19 vaccine (Sinovac/Butantan), in a 2-dose homologous vaccination regimen with a 28-day window and to recombinant covid-19 vaccine (Janssen) in a single dose regimen.

In spite of the availability of different vaccines, worldwide production has not been enough to overcome the mass vaccination demand, especially if taking into consideration the need of vaccines for second dose homologous regimen and an eventual third dose vaccine boost. Thus, it is paramount to understand flexible immunization regimens so as to generate additional data and cater to public health strategies to control this disease.

Studies using two-dose inactivated vaccines show an early drop in antibody titers and a third dose may be required to maintain or reinstall immune protection. Therefore, in addition to vaccination regimens involving a heterologous (with different vaccine types)[12] two-dose regimen became essential to obtain data on more flexible vaccine regimens, where the third dose different from the first homologous two-dose vaccine regimen broadly used to date. The purpose of this study is to assess the immunogenicity and safety of homologous (adsorbed inactivated covid-19 vaccine (Sinovac/Butantan) and heterologous third booster vaccine dose (recombinant covid-19 vaccine, AstraZeneca/Fiocruz; mRNA covid-19 vaccine, Pfizer/Wyeth or recombinant covid-19 vaccine, Janssen) in subjects previously vaccinated with two doses of adsorbed inactivated covid-19 vaccine (Sinovac/Butantan)

Primary immunogenicity objective	Endpoints	Estimations
• To assess the humoral immune response of a homologous or heterologous booster regimen (third dose) in subjects previously vaccinated with two Sinovac/Butantan doses, as per the following groups (overall; age stratification 18-60 versus > 60 years old):<br><br>• **Group 1**: (N=295) - Sinovac/Butantan (homologous)<br>• **Group 2:** (N=295) – AstraZeneca/Fiocruz (heterologous)<br>• **Group 3:** (N=355) - Pfizer/Wyeth (heterologous)<br>• **Group 4:** (N=295) – Janssen (heterologous)	• Anti-Spike IgG antibody titers of SARS-CoV-2 by ELISA methodology and by other assays for antibody detection on Day 28.	• GMTs;<br>• GMFRs (after third dose boost versus Day 1);
**Secondary Immunogenicity Objectives**		
• To assess neutralizing immune response of a homologous or heterologous booster regimen (third dose) against wild-type strain as per the following groups (overall; age stratification 18-60 vs. > 60 years old):<br>○ **Group 1**: (N=295)- Sinovac/Butantan (homologous)<br>○ **Group 2:** (N=295) – AstraZeneca/Fiocruz (heterologous)	• Neutralizing titer (NT) against SARS-CoV-2 Wuhan on Day 28 (per subgroup)	• GMTs;<br>• GMFRs (after third dose boost versus Day 1);

○ **Group 3:** (N=355) - Pfizer/Wyeth (heterologous)<br>○ **Group 4:** (N=295 – Janssen (heterologous)		
• To assess humoral immunity 6 months after primary vaccination with two Sinovac/Butantan doses (overall; age stratification 18-60 *vs* > 60 years old).	• Anti-Spike IgG antibody titers (by ELISA and by other assays for antibody detection) and NT against SARS-CoV-2 Wuhan on Day 1	• GMT
• To assess neutralizing immune response against wild-type strain and against concern variants of homologous or heterologous third booster dose regimens in subjects previously immunized with Sinovac/Butantan (2 doses).	• Neutralizing titer (NT) against SARS-CoV-2 (alpha, beta, gamma and delta variants) on Day 28 (per subgroup).	• GMTs<br>• GMFRs (after third dose boost versus Day 1);

Secondary Safety Objective		
• To assess the reactogenicity and safety of third booster dose in a homologous and heterologous vaccination regimen in subjects previously immunized with two Sinovac/Butantan doses.	• Occurrence of local and systemic AEs reported within 7 days after study vaccination (per subgroup);<br>• Occurrence of unsolicited AEs reported within 28 days after study vaccination (per subgroup);<br>• Occurrence of SAEs, MAAEs and AESIs within 28 days after study vaccination (per subgroup);	• Rate of subjects with solicited local and systemic AEs;<br>• Rate of subjects with unsolicited AEs;<br>• Rate of subjects with SAEs;<br>• Rate of subjects with MAAEs;<br>• Rate of subjects with AESIs;<br>per safety subgroup (FAS).
**Exploratory Safety Objective**		
• Documented confirmed SARS-CoV-2 symptomatic infection	• Occurrence of confirmed symptomatic cases during the study period	• Number of confirmed SARS-CoV-2 symptomatic cases;<br>• Severity of confirmed cases of SARS-CoV-2 infection (WHO scale).

Abbreviations: AE, adverse event; AESIs, adverse event of special interest; ELISA, enzyme-linked immunosorbent assay; FAS, full analysis group; GMFR, geometric mean of the titer; GMT, geometric mean titer; LLoQ, lower limit of quantification; MAAE, medically-attended adverse event; PPS, per protocol set; SAE, serious adverse event;
Note: Missing data will not be imputed for all immunogenicity and safety analyses.

Study design

This is a phase 4, single-blind, randomized, controlled, multicenter study to assess the immunogenicity and safety of a third heterologous booster dose with recombinant covid-19 vaccine (AstraZeneca/Fiocruz), mRNA covid-19 vaccine (Pfizer/Wyeth) or recombinant covid-19 vaccine (Janssen)in subjects primed with two doses of Sinovac/Butantan vaccines, compared to a third homologous booster dose of adsorbed inactivated covid-19 vaccine (Sinovac/Butantan) in adults.

The study will be performed in people receiving two doses of Sinovac/Butantan vaccine within a window between doses of 14-28 days (+ 7 days), 182 days (± 30 days) after the second dose prior to enrollment. The four study groups who will receive the third dose will be assigned as follows:

Subjects (N=1240)

- **Group 1**: (N=295) - Sinovac/Butantan (homologous)
- **Group 2:** (N=295) – AstraZeneca/Fiocruz (heterologous)
- **Group 3:** (N=355) - Pfizer/Wyeth (heterologous)
- **Group 4:** (N=295) – Janssen (heterologous)

The study design is displayed in Figure 1 and the schedule of activities is displayed in Table 1.

Age disposition: Each group will be divided into two subsets as per age (considering the margin of 4% for more or less to be distributed among subgroups):

- **Subset A**: between 18-60 years; approximately 150 per group.
- **Subset B**: >60 years old; approximately 150 per group.

Study groups: There will be four study groups, divided into 2 subsets each.

Study Group Assignment: Randomization (5:5:6:5) will be applied for subjects (**Table S1)** to account for the different number of doses per vial for different vaccines and short shelf life.

Table S1 Subject Disposition

Primary Vaccination (1st and 2nd Doses)	Booster Dose (3rd Dose)	Group (N)							
		1A	1B	2A	2B	3A	3B	4A	4B
Sinovac/Butantan	Sinovac/Butantan	147	148						
Sinovac/Butantan	AstraZeneca/Fiocruz			147	148				
Sinovac/Butantan	Pfizer/Wyeth					177	178		
Sinovac/Butantan	Janssen							147	148

Vaccination Schedule: All subjects will be given a third booster dose with one of vaccines Sinovac/Butantan, AstraZeneca/Fiocruz, Pfizer/Wyeth or Janssen on Day 1.

Study visits: There are two planned study visits on Days 1 and 28.

Safety contact: Participants will receive a contact by telephone or by any electronic means from the site staff (e.g., text messages – SMS, WhatsApp, email) between days 7 and 15 after Visit 1, in order to be asked about their health status, possible complications, symptoms of COVID-19, hospitalizations, and participant's diary completion.

Number of samples/blood tests: Two blood draws; one on Day 1 and the second on Day 28 will be obtained in all subjects.

Length of Participation: Approximately 30 days per subject.

Summarized inclusion/exclusion criteria: male and female subjects aged 18 years old or more, who were previously given two Sinovac/Butantan vaccine doses. For the detailed list of inclusion and exclusion criteria, see Sections 5.1 and 5.2.

Study vaccines/comparators:

- Adsorbed covid-19 vaccine (inactivated) ─ Sinovac/Butantan: is an inactivated COVID-19 vaccine developed by Sinovac in partnership with Instituto Butantan. Each 0.5-mL dose contains 600 SU of inactivated SARS-CoV-2 virus, storage at 2 to 8ºC.
- Recombinant covid-19 vaccine –AstraZeneca/Fiocruz: contains chimpanzee adenovirus codifying Spike SARS-CoV-2 glycoprotein, each 0.5-mL dose contains 10 x 10^{10} viral particles; storage between 2 and 8ºC.
- mRNA covid-19 vaccine - Pfizer/Wyeth: is a mRNA vaccine (incorporated into lipid nanoparticles), each 0.3 mL contains 30 µg of SARS-CoV-2 Spike (S) protein messenger RNA; storage between 2 and 8ºC (up to 31 days), -25 to ─15ºC (up to 14 days), -80 to ─60ºC (6 months).
- Recombinant covid-19 vaccine - Janssen: contains Adenovirus 26 codifying SARS-CoV-2 Spike glycoprotein (Ad26.COV2-S), at least 8.3 x 10^{10} infectious units (Inf. U) in a 2.5-mL multidose vial, used as a single dose of 0.5 mL containing 5 x 10^{10} viral particles; storage between 2 and 8ºC (4.5 months), -25 to -15ºC (24 months).

Vaccine administration route: Intramuscular (IM).

Immunogenicity assessments:

Humoral immune responses will be assessed by using the following assays:

- ELISA and other immunogenicity assays for detection of IgG against Spike (S) protein of SARS-CoV-2.
- Viral neutralization assay (VNA, neutralizing SARS-CoV-2 activity using vaccine homologous wild-type strain).
- VNA, SARS-CoV-2 neutralizing activity using vaccine heterologous wild-type strain(s), including, but not limited to, alpha, beta, gamma and delta variants.

VNAs and ELISAs results and any other antibody detection assay will be expressed as:

- Geometric mean of the titer (GMT, IU/mL).
- Geometric mean increase rate (GMFR) as increases in post-vaccination titer compared to pre-vaccination titer.

Safety assessments:

The following safety data will be collected:

- All subjects will be monitored for 30 minutes ± 10 after each study injection for any immediate adverse reactions.
- Local and systemic unsolicited adverse events (AEs) will be recorded daily within 7 days after the study vaccination:
  - Local reactions: pain, redness and swelling at the injection site.
  - Systemic AEs: fatigue, headache, myalgia, arthralgia, loss of appetite, nausea, chills and fever.
- Any unsolicited AEs will be collected within 28 days after study vaccination in all subjects.
- Any medically-attended adverse event (MAAEs), serious AEs (SAEs), AEs with special interest (AOSIs) and AEs leading to study early termination will be reported throughout the study period. This data will be collected at visit 2 to the site and telephone contacts or electronic means (contacts via SMS message or WhatsApp app). Any diagnosed episodes of SARS-CoV-2 infections will be reported as part of MAAEs; no active surveillance for SARS-CoV-2 infection is planned in this study.

Statistical considerations:

Sample size:

For the immunogenicity objective, the study will need to recruit at least 124 evaluable subjects per subgroup. The randomization ratio will be 5:5:6:5 to allow for vaccine vials with different numbers of doses and a short shelf life. Therefore the total randomization will be 1240 (295:295:355:295) which also allows for some loss to follow up.

Primary immunogenicity analysis:

Primary endpoints for immunogenicity will be assessed by GMT, GMFR and SCR by ELISA methodology and other antibody detection assay for Spike (S) protein on Days 1 and 28 for each vaccine regimen as per-protocol set (PPS), which includes all subjects receiving the correct vaccination and with no major protocol deviations, and FAS if more than 5% of subjects are excluded from the PPS.

Safety analysis:

Safety analysis (FAS) will be assessed by reactogenicity summaries (solicited AEs) and unsolicited AEs/SAEs/MAAEs/AESIs per safety analysis group, which includes all subjects given the third study vaccine dose.

- Reactogenicity will be assessed by frequencies and percentages of subjects who experienced each solicited AE along with 95% CIs for each symptom by any seriousness (Grades 1 to 4, except for fever) for 7 days after vaccination.
- For unsolicited AEs, SAEs, MAAEs and AESIs
  - Frequencies and percentages of subjects reported with at least one AE after 28 days of vaccination will be summarized by vaccine group, along with 95% CIs by Clopper-Pearson method.
  - Frequencies and percentages of subjects reported with at least one SAE, MAAE and AESI will be summarized throughout the study per vaccine group and 95% CIs by Clopper-Pearson method.

Missing data will not be imputed.

1.1 Study design

The study design (booster regimen of homologous vs, heterologous third dose) is shown in figure 1.

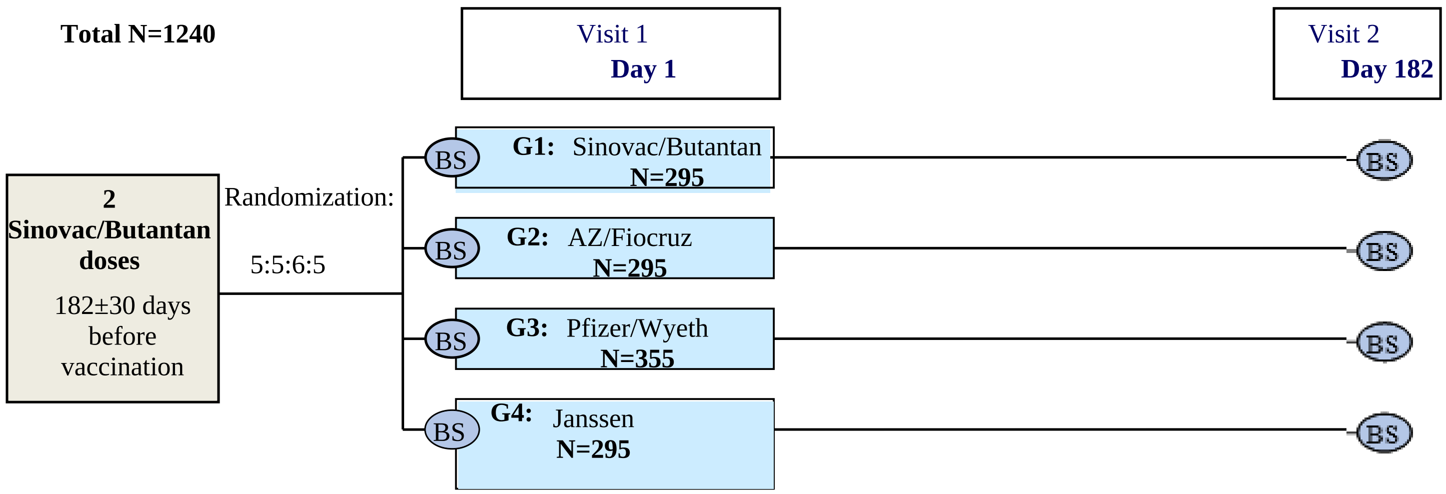

Figure 1 Study Design

Abbreviation: BS: blood sample.

1.2 Schedule of activities

The schedule of activities (SoA) is shown in Table 1.

Table 1 Schedule of Activities

Visit	Visit 1	Contact	Visit 2
**Timepoint in regards to vaccination**	**3rd dose**	**7 – 15 days**	**3rd dose +28d**
**Study day**	**D1**		**D28**
**Visit window**	**-**		**-7d/+7d**
Informed Consent Form[a]	X		
Demographics	X		
Medical history, previous medications and vaccines and against Covid-19	X		
Physical exam[b]	X (if required)		X (if required)
Vital signs[c]	X		X (temperature)
Pregnancy Test[d]	X		
Inclusion and exclusion criteria	X		
Randomization	X		
Vaccination	X		
30 minutes of post-vaccination observation[e]	X		
Daily card[f]	X		
Solicited AE report[g]			X
Unsolicited AE report[g]			X
SAE report			X
AESI report			X
MAAE report			X
Concomitant medication report			X
Blood sample draw for IgG Anti-Spike immunology	X[i]		X
Safety contact		X	
Subject unblinding			X
Study completion			X

Abbreviations: AE, adverse event; AESI, adverse event of special interest; d and D, day(s); MAAE, adverse event with medical care; SAE, serious adverse event; IME, important medical event; SARS-CoV-2, serious acute respiratory syndrome by coronavirus 2.

Notes:

[a] The informed consent process and pre-vaccination procedures (physical exam, vital signs and pregnancy test, if applicable) may be performed within 3 days prior to Day 1. In case the subject experiences a mild disease preventing their participation in the study, they may be screened once again for one time.

[b]Physical exam should be performed by a qualified healthcare professional as per local rules, at medical discretion. See Section 8.3 for physical exam components per visit.

[c] See Section 8.3 for details on vital signs collected.

[d] A urine pregnancy test will be performed in women at childbearing potential prior to vaccination (see Section 8.4).

[e]Any AEs reported within 30 minutes after vaccination should be reported in the electronic case report form (eCRF).

[f]Daily card provision and training on Day 1 visits. Daily card review and check with the subject on Day 28.

[g] Solicited symptoms assessment will be performed within 7 days after vaccination and unsolicited AEs Visit 1 (Day 1) from Visit 2 (Day 2) (see Section 8.9). This will include review of daily card entries with the subject by the study doctor and/site team at Visit 2.

[i]To be collected prior to vaccination.

At each study contact, study subjects will be asked on a COVID-19 history and any positive SARS-CoV-2 test results. In case a study subject develops suspected disease or symptoms of coronavirus disease 2019 (COVID-19), a diagnostic investigation should be made. This test includes nasal, nasopharyngeal or oropharyngeal swab collection for SARS-CoV-2 reverse transcriptase polymerase chain reaction (RT-PCR) and other diagnostic procedures as per the latest national guidance.

2.0 INTRODUCTION

The purpose of this study is to assess the immunogenicity of a booster dose using different anti-COVID-19 vaccines in subjects previously vaccinated with two Sinovac/Butantan doses. Comparison will be made in groups vaccinated with a homologous booster dose using Sinovac/Butantan or a heterologous booster dose (using AstraZeneca/Fiocruz, Pfizer/Wyeth or Janssen vaccines).

Background

Coronaviruses have positive sense single-strand RNA genome. Around a quarter of its genome codifies structural proteins, Spike (S) glycoprotein, envelope (E), membrane (M) and nucleocapside (N). In the virus causing coronavirus disease 2019 (COVID-19) referred to as SARS-CoV-2, Spike protein has a role in binding to the receptor, mediating the virus entry into host cells by recognizing angiotensin converting enzyme 2 (ACE2)[1,2]. COVID-19 is a highly infectious disease that has spread rapidly worldwide in the end of 2019. In January 2020, the World Health Organization (WHO) has declared COVID-19 outbreak as an international concern public health emergency.

To date, there is no treatment for COVID-19, however, strategies of mass vaccination have impacted the pandemics in countries such as Israel and the US. In spite the imminent success with vaccination, several countries have already experienced or are experiencing phenomena of "second" and "third waves" of infection, which may lead to the emergence of genetic SARS-CoV-2 variants, that, on their turn, may impact pandemics control. In this setting, genetic variants are divided into three categories. A Variant of Interest (VOI) refers to that related to changes in binding to cell receptor. In these variants, neutralization by antibodies generated in cases of infection or previous vaccination are seen to be decreased, and has a decreased efficacy to treatments, a potential diagnostic impact or expected increase in disease transmissibility and seriousness. A Variant of Concern (VOC) is related to the increased transmissibility and decreased neutralization ability by antibodies, as well as the decreased efficacy of treatments or vaccines. Last, a variant of high consequence (VHC) is used to describe those with clear evidence that prevention measures or medical countermeasures (MCMs) have no effect when compared to previous circulant variants.

COVID-19 pandemics has overwhelmed healthcare systems worldwide and the development of vaccines has an essential role to control the pandemics and avoid the collapse of healthcare systems. In this setting, the development of vaccine regimens will be paramount. In Brazil, ANVISA granted authorization for AstraZeneca/Fiocruz vaccine in a 2-dose vaccination regimen with a window of 28 to 84 days. Moreover, the authorization was also granted for Pfizer/Wyeth vaccine in a 2-dose vaccination regimen, with a window of 21-84 days. Emergency use authorization (EUA) was granted to Sinovac/Butantan vaccine in a 2-dose regimen with a 28-day window and to Janssen in a single dose regimen. Other vaccines using different platforms are expected to be approved for use against COVID-19, and most should be approved in a 2-dose vaccination regimen.

2.1 Study rationale

Vaccines for COVID-19 were shown to confer protection against infection by SARS-CoV-2[3,4,5]. However, the appearance of new SARS-CoV-2 virus variants[6] and the reported decrease in antibody titers in vaccinated subjects[7] are extremely concerning. Moreover, in view of the shortage of vaccine provision and evidence of rare, but fortuitously serious adverse effects after vector-based vaccines[8], there is great interest in the development of alternative vaccination regimens. In this setting, a heterologous vaccine regimen (that using two different vaccines) for COVID-19 arises as an accessible strategy that may mitigate supply shocks of shortage of vaccine availability[9,10].

Currently, searching clinical trial registry platforms (clinicaltrials.gov; CTRI and ChiCTR), eight international clinical trial records are found assessing a third vaccine dose, both in an interchangeable regimen and as a third homologous dose to the initial vaccine regimen. The following combinations are being tested in these studies: Sinovac/Sinovac/Sinovac; Sinovac/Sinovac/Cansino; Moderna/Moderna/Moderna; Pfizer/Pfizer/Pfizer; Sinopharm/Sinophar/Sinophar; West China/West China/West China; Zydus Cadila/Zydus Cadila/Zydus Cadila and Anhui Zhifei/Anhui Zhifei/Anhui Zhifei.

A vaccine protocol using a heterologous booster dose may lead to a higher increase in immune response than that reached in a conventional vaccine protocol or even in a protocol using a homologous booster dose[11]. The first immunogen is believed to activate memory cells, while the second would be responsible for its expansion[12]. Moreover, given the challenges to immunize large populations, especially in regards to supply, shortage of goods and possible vaccine mixtures, it is crucial to develop more flexible vaccination protocols, where doses are used with different authorized vaccines. In this setting, the information from this study will generate evidence-based data for the national and international scientific community as for vaccination and public health strategies that are extremely important to fight COVID-19.

2.1.1 Scientific rationale for the study design

Currently in Brazil, there are vaccines authorized for emergency and definite use, most of them using a two-dose vaccine regimen. Due to the decreased immunity with antibody titer reduction, and also due to the appearance of variants of concern for which high antibody titers are required, there is an urgent need to analyze antibody persistence and the booster effect of a third dose.

From epidemiological researches performed in countries which mainly used inactivated vaccines, in spite of the high early vaccination coverage, there were marked increases of new cases with the increase in second dose window[13].

A third dose may be given both as a homologous and heterologous booster. Studies with an initial heterologous vaccine regimen (first dose with vaccine A and second dose with vaccine B) seem to be immunologically superior.

In this study, subjects who were given two doses of Sinovac/Butantan vaccine prior to entering the study will receive a new booster dose (3rd dose) of Sinovac/Butantan vaccine (homologous approach) or recombinant covid-19 vaccine (Fiocruz/AstraZeneca), mRNA covid-19 vaccine (Pfizer/Wyeth) or recombinant covid-19 vaccine (Janssen) (heterologous approach). The safety and immunogenicity of a homologous or heterologous booster dose will be assessed and data obtained in this study may help in the development of additional protocols adding a third dose for vaccination against COVID-19.

2.2 Risk/benefit assessment

2.2.1 Risk assessment

The following section describes the risk assessment and mitigation strategy for this study protocol (Table 2):

Table 2 Major risks and mitigation strategy

Potential of major risk	Data/Rationale for risk	Mitigation strategy
**General Risks**		
Hypersensitivity, including allergic reactions, such as anaphylaxis	Acute allergic reactions, such as an anaphylactic event, may occur by dosing with any vaccine. These are serious, but rare occurrences, estimated in the range of 1 to 10 cases per million vaccinations, depending on the studied vaccine.[11]	The onset of vaccine-related allergic symptoms usually occurs within a few minutes. To be able to treat a subject with an allergic reaction to the vaccination, the subject should remain in observation (followed visually; no specific procedure) at the study site for 30 minutes after vaccination, with medical treatment promptly available, if required.
Reaction at the injection site	IM vaccination usually triggers a transient self-limited local inflammatory reaction. This may usually include pain, redness and swelling at the injection site.	Local solicited AEs will be monitored up to 7 days after vaccination.
**Risks Related to Study Procedures**		
Venous puncture (blood sample)	Pain or bruising where blood is drawn.	Blood samples will be drawn by trained medical personnel; subjects will remain under medical observation after venous puncture to complete study-specific procedures.
Syncope	Syncope (fainting) may occur after or even prior to any blood draw:, as a psychogenic or vasovagal response to needle injection.	All subjects will remain under observation after venous puncture, until completing the applicable study visit.

Abbreviations: AE, adverse event; DSMB, data safety monitoring committee.

2.2.2 Benefit Assessment

Study subjects may benefit directly from participation in this study if vaccinations induce or increase a protective immune response against SARSCoV2 when and if these subjects are subsequently exposed to the virus. Subjects will obtain some information on their general health, including their information status by SARSCoV2, a result from the screening history and medical test. Subjects with a condition not diagnosed previously and who require more medical attention will be properly referred for additional investigation and treatment, with their permission. Their contribution to this study is expected to support even more with information that will generate evidence-based data for the national and international scientific community as for vaccination and public health strategies that are extremely important to fight COVID-19.

2.2.3 Overall risk/benefit conclusion

Taking into consideration the measures to be implemented to minimize risks for subjects in this study, potential and/or identified known risks related to vaccines formulations of adsorbed (inactivated) covid-19 vaccine Sinovac/Butantan, recombinant covid-19 vaccine (Fiocruz/AstraZeneca), mRNA covid-19 vaccine (Pfizer/Wyeth) or recombinant covid-19 vaccine (Janssen) are justified by the benefits foreseen for humans to prevent SARS CoV-2 infection and COVID-19.

This study will be carried out in compliance with the protocol, International Conference on Harmonization (ICH) Good Clinical Practices (GCP) and applicable regulatory requirements. The study aspects related to study vaccines will comply with Good Manufacturing Practices (GMP) requirements.

More detailed information on known and expected risks and benefits and reasonably expected AEs from the vaccine may be found in the leaflets of vaccines used.

3.0 OBJECTIVES, ENDPOINTS AND ESTIMATIONS

The study objectives, endpoints and estimations are listed in Table 3.

Table 3 Objectives, endpoints and estimations

**Primary immunogenicity objective**	**Endpoints**	**Estimations**
• To assess the humoral immune response of a homologous or heterologous booster regimen (third dose) in subjects previously vaccinated with two Sinovac/Butantan doses, as per the following groups: (overall; age stratification 18-60 *vs.* >60 years):	• Anti-Spike IgG antibody titers on Day 28 measured by ELISA methodologies and by other assays for antibody detection.	• GMTs;<br>• GMFRs (after third dose boost versus Day 1);
**Secondary Immunogenicity Objectives**		
• To assess neutralizing immune response of a homologous or heterologous booster regimen (third dose) against wild-type strain as per the following groups (overall; age stratification 18-60 vs. > 60 years old)	• Neutralizing titer (NT) against SARS-CoV-2 Wuhan on Day 28 (per subgroup)	• GMTs;<br>• GMFRs (after third dose boost versus Day 1);
• To assess humoral immunity persistence 6 months after primary vaccination with two Sinovac/Butantan doses (overall; age stratification 18-60 *vs* > 60 years old).	• Anti-Spike antibody titers (by ELISA and by other assays for antibody detection) and NT against SARS-CoV-2 Wuhan on Day 1	GMTs
• To assess neutralizing immune response against wild-type strain and against concern variants of homologous or heterologous third booster dose regimens in subjects previously immunized with Sinovac/Butantan (2 doses).	• Neutralizing titer (NT) against SARS-CoV-2 (alpha, beta, gamma and delta variants) on Day 28 (per subgroup).	• GMTs<br>• GMFRs (after third dose boost versus Day 1);

Secondary Safety Objective		
• To assess the reactogenicity and safety of third booster dose in a homologous and heterologous vaccination regimen in subjects previously immunized with two Sinovac/Butantan doses.	• Occurrence of local and systemic AEs reported within 7 days after study vaccination (per subgroup);<br>• Occurrence of unsolicited AEs reported within 28 days after study vaccination (per subgroup);<br>• Occurrence of SAEs, MAAEs and AESIs throughout the study period in all groups.	• Rate of subjects with solicited local and systemic AEs;<br>• Rate of subjects with unsolicited AEs;<br>• Rate of subjects with SAEs;<br>• Rate of subjects with MAAEs;<br>• Rate of subjects with AESIs;<br>per safety subset (FAS).
**Exploratory Safety Objective**		
• Documented confirmed SARS-CoV-2 symptomatic infection	• Occurrence of confirmed symptomatic cases during until day 28.	• Number of confirmed SARS-CoV-2 symptomatic cases;<br>• Severity of confirmed cases of SARS-CoV-2 infection (WHO scale)

Abbreviations: AE, adverse event; AESIs, adverse event of special interest; ELISA, enzyme-linked immunosorbent assay; FAS, full analysis group; GMFR, geometric mean fold rise; GMT, geometric mean of the titers; LLoQ, lower limit of quantification; MAAE, medically-attended adverse event; PPS, per protocol set; SAE, serious adverse event.
Note: Missing data will not be imputed for all immunogenicity and safety analyses.

4.0 STUDY DESIGN

A phase 4, single-blind, randomized, controlled, multicenter study to assess the immunogenicity and safety of a third homologous and heterologous booster dose vaccination using Sinovac/Butantan, Fiocruz/AstraZeneca, Pfizer/Wyeth, Janssen in subjects previously vaccinated with two Sinovac/Butantan vaccine doses.

4.1 Overall design

Subject disposition for the study is shown in table 4. A total of 1240 subjects is planned.

Table 4 Subject Disposition

		Group (N)							
**Primary Vaccination (1st and 2nd Doses)**	**Booster dose (3rd Dose)**	**1A**	**1B**	**2A**	**2B**	**3A**	**3B**	**4A**	**4B**
Sinovac/Butantan	Sinovac/Butantan	147	148						
Sinovac/Butantan	AstraZeneca/Fiocruz			147	148				
Sinovac/Butantan	Pfizer/Wyeth					177	178		
Sinovac/Butantan	Janssen							147	148

Randomization (5:5:6:5) will be applied for subjects from groups 1 to 4.

The study design is provided in Figure 1; the SoA is provided in Table 1.

4.2 End of study definition

The end of study is defined as the date of the last subject last visit in the study or last scheduled procedure shown in the SoA (Table 1) for the last study subject.

5.0 STUDY POPULATION

5.1 Inclusion criteria

Subjects are eligible to be enrolled in the study only if all of the following criteria apply:

1. Males or females aged ≥18 years old.
2. Subjects willing and able to comply with the study procedure.
3. Subjects willing and able to provide informed consent prior to screening.
4. Subjects receiving two Sinovac/Butantan vaccine doses, 182 days (±30 days) prior to enrollment in this study.
5. Female subjects are eligible to take part in the study if not pregnant, puerperal or nursing.

5.2 Exclusion criteria

Subjects will be excluded from the study if any of the following criteria apply:

1. Subjects with fever >37.5 °C (axillary) or any acute disease at baseline (Day 1) or within the 3 days prior to randomization. Febrile subjects with mild diseases may be enrolled at the investigator's discretion.
2. Subjects with a history of COVID-19, laboratory confirmed.
3. Subjects with a history of serious vaccine-related adverse reaction or serious allergic reaction (e.g., anaphylaxis) to any study vaccine component, as described in the last summary of product characteristics for Sinovac/Butantan, Fiocruz/AstraZeneca, Pfizer/Wyeth, Janssen.
4. Subjects with a known bleeding disorder that, in the investigator's opinion, would contraindicate intramuscular injection.
5. Subjects with any progressive or serious neurological disorder, seizure disorder or history of Guillian-Barré syndrome.
6. Subjects given treatment with immunosuppressant therapy within the last 90 days, including cytotoxic agents or systemic corticosteroids or planned receipt during the study period. If a short-term cycle of immunosuppressant systemic corticosteroid dose has been used to treat acute disease, the subject should not be enrolled in the study until corticosteroid therapy has been discontinued for at least 15 days prior to the first study vaccination. In case the subject has been on an immunosuppressant dose of a depot, intramuscular or intra-articular corticosteroid, 60 days should be waited for their enrollment in the study. Inhaled/nebulized, intra-articular, intrabursal or topical (skin or eyes) corticosteroids are allowed.
7. Subjects with autoimmune diseases, other than: Hashimoto thyroiditis, vitiligo, psoriasis, discoid lupus and the like; HIV-positive subjects and/or in treatment for HIV;

8. Subjects given any other investigational product within the 30 days prior to Day 1 or who intend to take part in another clinical trial at any time during this study conduction.
9. Subjects given any other licensed vaccine within 14 days prior to enrollment in this study or who plan to receive any vaccine up to 28 days after vaccination.
10. Subjects given treatment with Rituximab or any other anti-CD20 monoclonal antibody within 9 months prior to Day 1 or planned during the study period.
11. Administration of intravenous immunoglobulins and/or any blood products within 3 months prior to enrollment or planned dosing during the study period.
12. Subjects with any condition that, in the investigator's opinion, could interfere with the status primary objectives or represent an additional risk for the subject.
13. Any other vaccine for Covid-19 other than two Sinovac/Butantan doses.

5.3 Previous and concomitant medications

The use of previous and concomitant medications should be recorded in the electronic case report form (eCRF).

- Previous drugs, vaccines and blood products described in the exclusion criteria should be recorded in the eCRF, when given, including:
  - Every drug and vaccine dosed at Visit 1 (vaccination day) up to 28 days;
- Every drug meeting the reporting criteria (including over-the-counter or prescribed drugs and/or herbal supplements) should be recorded in the eCRF along with:
  - Reason for use.
  - Dosing dates, including start and end dates.
  - Dosing information, including dose and frequency.
- The use of antipyretics (e.g., paracetamol) will be allowed as a treatment for fever and pain and other post-vaccination reactions; subject should not be encouraged to use prophylactic antipyretics. The medical monitor should be contacted in case there is any doubt on concomitant or previous therapy.

5.4 Forbidden Drugs

The use of an excluded medication/therapy is a protocol violation: and should be recorded in the eCRF. The use of an excluded drug does not require subject withdrawal from the study.

The following drugs are forbidden:

- Rituximab or any other anti-CD20 monoclonal antibody within 9 months prior to Day 1 or during the study period.
- Immunosuppressant therapy, including cytotoxic agents or systemic corticosteroids, within the previous 90 days to Day 1 or during the study period
- Intravenous immunoglobulins and/or any blood products within the 3 months prior to Day 1 or during the study period

- Receipt of any investigational product within 30 days prior to Day 1 or during the study period.
- receipt of any authorized or investigational vaccine for COVID-19 prior to Day 1 (except for two Sinovac/Butantan doses) or during the study.

5.5 Screening failures

Screening failures are defined as subjects consenting to take part in the clinical trial, but do not meet all eligibility criteria and are not subsequently randomly assigned to a study arm. A minimum set of screening failures information is required to assure transparent reports of subjects with screening failures so as to meet publications requirements of the Consolidated Standards of Trial Reports (CONSORT) and to reply to regulatory authorities' queries. Minimum information includes the reason for screening failure.

6.0 VACCINES/COMPARATORS

All vaccines related to this study should be stored separately from other vaccines and drugs in a safe place with temperature monitoring. All vaccines related to this study should be checked for expiration date prior to use. Expired study vaccines should not be given to subjects.

Adsorbed covid-19 vaccine (inactivated) – Sinovac/Butantan: is an inactivated COVID-19 vaccine developed by Sinovac in partnership with Instituto Butantan. Each 0.5-mL dose contains 600 SU of inactivated SARS-CoV-2 virus, storage at 2 to 8ºC.

Contraindication:

- Allergic reactions to any components of this vaccine
- Patients with fever, acute disease and acute-onset of chronic diseases

Recombinant covid-19 vaccine –AstraZeneca/Fiocruz: contains chimpanzee adenovirus codifying Spike SARS-CoV-2 glycoprotein, each 0.5-mL dose contains 10 x 10^{10} viral particles; storage between 2 and 8ºC.

Contraindication:

- Hypersensitivity to the active substance or any vaccine excipient.
- Do not give to subjects with a history of serious allergic reaction (e.g., anaphylaxis) to any vaccine component.

mRNA covid-19 vaccine - Pfizer/Wyeth: is a mRNA vaccine (incorporated into lipid nanoparticles), each 0.3 mL contains 30 µg of SARS-CoV-2 Spike (S) protein messenger RNA; storage between 2 and 8ºC (up to 31 days), -25 to –15ºC (up to 14 days), -80 to –60ºC (6 months).

Contraindication:

- Hypersensitivity to the active substance or any vaccine excipient.
- Do not give to subjects with a history of serious allergic reaction (e.g., anaphylaxis) to any vaccine component.
  - Recombinant covid-19 vaccine - Janssen: contains Adenovirus 26 codifying SARS-CoV-2 Spike glycoprotein (Ad26.COV2-S), at least 8.3 x 10^{10} infectious units (Inf. U) in a 2.5-mL multidose vial, used as a single dose of 0.5 mL containing 5 x 10^{10} viral particles; storage between 2 and 8ºC (4.5 months), -25 to -15ºC (24 months).

Contraindication:

- Hypersensitivity to the active substance or any vaccine excipient
- Do not give to subjects with a history of serious allergic reaction (e.g., anaphylaxis) to any vaccine component.

The investigator should record all study vaccine injections provided to subjects.

All dosings should be performed at the study site by duly trained personnel.

The medical team is in charge of ongoing subjects' safety and well-being while in the study site. There is a physician at the site during normal operation hours and medical advice is available by telephone 24 hours a day. Moreover, if required, the medical team may contact other physicians on call or public emergency services in the event of a serious medical event.

More details on preparation, handling, storage are provided in product leaflets.

WARNINGS AND PRECAUTIONS

Only subjects enrolled in the study may receive study vaccines and solely the authorized study team may provide or dose study vaccines. The availability of facilities and personnel experienced with anaphylaxis treatment and resuscitation and with treatment of other medical emergencies will be assured.

6.1 Subject randomization and blinding

This will be a single-blind study, i.e., blind for the subject, who will receive information on the vaccine received at Visit 2.

After signing the Informed Consent Form (ICF), each subject will receive a screening number as per the screening order. On Day 1, after confirming the eligibility, a subject number will be assigned and subjects will be assigned to treatment by using an interactive response technology (IRT).

Due to visual differences among study vaccines, subjects will remain blinded prior to receiving the study vaccination.

6.2 Study vaccine data recording

The investigator records all study vaccine injections provided to subjects.

The prescribed dosing, time and mode of administration cannot be changed. Any deviations from the intended regimen should be recorded in the eCRFs.

All dosings should be performed at the study site by duly trained personnel.

The medical team is in charge of ongoing subjects' safety and well-being while in the study site. There is a physician at the site during normal operation hours and medical advice is available by telephone 24 hours a day. Moreover, if required, the medical team may contact other physicians on call or public emergency services in the event of a serious medical event.

7.0 SUBJECT WITHDRAWAL FROM THE STUDY

7.1 Early withdrawal

The primary reason related to premature study discontinuation will be documented in the eCRF:

- Subject's decision;
- Adverse Event;
- Loss of follow-up;
- Study termination by the sponsor;

In case a subject not meeting the inclusion criteria is inadvertently enrolled, this subject should be discontinued from the study and the Sponsor or person assigned by the Sponsor should be contacted. An exception may be granted in rare circumstances for which there is a compelling safety reason to allow the subject to continue and the Investigator should document it.

7.2 Subject withdrawal

A subject may withdraw from the study at any time at their own request, or may be withdrawn at any moment at the investigator's discretion for safety, behavior, compliance of administrative reasons.

If the subject withdraws the consent to disclose future information, the sponsor may retain and continue to use any data collected prior to said consent withdrawal.

If a subject withdraws from the study, they may request the destruction of all samples collected and not tested, and the investigator should document this in the study site study records and inform the sponsor representative.

See Table 1 for all data to be collected at study discontinuation and follow-up and any additional assessments that need to be completed.

7.3 Loss of follow-up

A subject will be deemed as loss of follow-up if they fail to return to visit 2 and cannot be reached from the study site.

The study site should try to contact the subject and reschedule the missed visit as soon as possible and advise the subject on the importance of keeping the assigned visit schedule and check whether the subject wishes or not and/or should continue in the study.

Before a subject is deemed as a loss of follow-up, the investigator or designee should make all efforts to regain contact with the subject (when possible, 2 telephone calls and/or electronic message, one registered letter to the last known subject's mailing address or local equivalent methods). These contact attempts should be documented in the subject's chart.

If the subject remains not accessible, they will be deemed as having withdrawn from the study.

8.0 STUDY ASSESSMENTS AND PROCEDURES

The following sections describe the study procedures and data to be collected. All procedures should be performed by qualified and trained personnel.

Protocol waivers are not allowed.

Safety issues should be immediately discussed with the sponsor after the occurrence or acknowledgment so as to determine whether the subject should continue or discontinue the study vaccine.

The contract research organization (CRO) will assure real time safety data assessments.

Compliance to study design requirements, including those specified in the SoA, is crucial and required to conduct the study.

All screening assessments should be completed and reviewed so as to confirm that potential subjects meet all eligibility criteria. The investigator will keep a screening log to record the details of all screened subjects and to confirm the eligibility or record the reasons for screening failure, as applicable.

The details of blood sample draw, biological sample handling and analysis will be shown in the laboratory manual. Repeated or unscheduled samples may be collected for safety reasons or for technical issues with the existing samples.

8.1 Informed Consent Form

The informed consent should be obtained prior to performing any protocol-driven procedure.

The informed consent form requirements are described in Appendix 2.

8.2 Demographics

Demographics to be collected on the subject will include age/date of birth (if applicable), sex, race (and ethnicity) and medical history, including the presence of any medical condition related to high risk of serious COVID-19.

Previous medications should be collected as specified in Section 5.3.

See Section 5.4 for details on forbidden drugs.

This data is recorded in the source document.

8.3 Physical Exam and Vital Signs

Vital signs will be obtained at visit 1 and physical exams should be performed, at the investigator's discretion, if deemed as required, directed to symptoms and/or vital signs, by a qualified healthcare professional as per the local practice and medical rules and as listed in the site delegation of responsibility log. Symptom-directed physical exam may be performed at visit 2, if required, and vital signs, temperature only.

This data is recorded in the source document and any abnormal values or events should be documented in the eCRF AE form. The treatment of any abnormality should be performed as per local medical practice and out of this study or through referral to a proper healthcare professional.

Prior to study vaccination, vital sign assessments (systolic/diastolic blood pressure, respiratory rate, body temperature and heart rate) will be measured.

8.4 Pregnancy Test

A urine pregnancy test will be performed for women at childbearing potential prior to vaccination on Day 1.

8.5 Inclusion and exclusion criteria

Only subjects who signed the ICF and meet all inclusion (Section 5.1) and exclusion (Section 5.2) criteria are eligible for randomization and to enter the study.

If a subject is not eligible for randomization, the investigator should record the main reason for subject screening failure and enrollment recording (Section 5.5).

8.6 Randomization

Randomization specification will be approved by the study sponsor statistician or designated person.

The vaccines have different shelf life and number of doses per vial. Therefore, randomization will be performed in blocks of 42 doses/6h period to minimize the potential for vaccine wastage.

Randomisation will therefore be done in a 5:5:6:5 ratio, stratified by age group and day of randomisation

The table below summarizes this information.

Vaccine	Expire Time after open vial	Number of doses/vial	Number of the vials per block of randomization (# of doses/randomization)
AstraZeneca/Fiocruz	48h	5	2 (10)
Sinovac/Butantan	8h	10	1 (10)
Pfizer/Wyeth	6h	6	2 (12)
Jassen	6h	5	2 (10)

8.7 Vaccination

All subjects will be monitored for 30 minutes (± 10 min) after the study injection for any immediate adverse reactions.

The instructions on study vaccine preparation and dosing are provided in the pharmacy manual.

Homologous or heterologous booster doses given in the study will be entered in a proper site form and the eCRF.

8.8 Immunogenicity assessments

Humoral immune responses will be assessed by using the following assays:

- ELISA and other immunogenicity assays for the detection of IgG against SARS-CoV-2 Spike (S) protein.
- VNA, SARS-CoV-2 neutralizing activity using vaccine heterologous wild-type strain, and alpha, beta, gamma and delta variants.

VNAs, ELISA assays and any other antibody detection assay results will be expressed as:

- Geometric mean of the titer (GMT, IU/mL).

- Geometric mean increase rate (GMFR) as increases in post-vaccination titer compared to Day 1 titer.

Immunogenicity assessments for this study are listed in Table 5.

Table 5 Immunogenicity assessments

Assay	Study endpoints	Sample	Days	Comment
ELISA and other assays for antibody detection	Primary endpoint	Serum	1 and 28	Quantification and isotyping of antibody response to S SARS-CoV-2 antigen by testing through ELISA methodology and other assays for antibody detection.
VNA (homologous vaccine and heterologous vaccine)	Secondary and exploratory endpoints	Serum	1 and 28	Functional antibody response functional assessment against SARS-CoV-2 S antigen by testing VNA with the 4 strains, Wuhan, alpha, beta, gamma, and delta.

Abbreviations: ELISA, enzyme-linked immunosorbent assay; viral neutralization assay.

Planned timepoints for all immunogenicity assessments are provided in the SoA (Table 1).

8.8.1 Blood volumes and serological tests

All subjects will provide up to 13 mL of blood at each visit, with a total volume of up to 26 mL in those who complete the study at day 28. The test plan is provided in table 6.

Table 6 Test plan

	Blood Volume	Frequency	Total volume
Other assays for antibody detection	3.5 - 5 mL	2	10 mL
VNA and ELISA	8 mL	2	16 mL
Maximum blood volume	13 mL	-	26 mL

Abbreviations: ELISA, enzyme-linked immunosorbent assay; viral neutralization assay

8.8.2 Biological sample retention and destruction

The details of biological sample collection, handling and analysis are shown in the laboratory manual. Repeated or unscheduled samples may be collected for safety reasons or for technical issues with the samples. Biological samples will be stored for up to 2 years after completing the study and will be destroyed after this period. The sponsor has implemented a system to protect subject's personal information so as to assure optimum confidentiality and standard procedures defined for sample collection, on-study storage, analysis and destruction.

8.9 Safety assessments

Safety assessments will include collection and recording of:

- Any spontaneous AEs within 30 minutes (± 10 min) after study vaccination;
- Solicited local and systemic AEs within 7 days after the study vaccination (Section 9.1.9):

  - Local reactions: pain, redness and swelling at the injection site.
  - Systemic AEs: fatigue, headache, myalgia, arthralgia, loss of appetite, nausea, chills and fever.
- Any unsolicited AEs within 28 days after study vaccination (Section 9.1.9).
- Any MAAE (Section 9.1.15), SAEs (Section 9.1.12), AESIs (Section 9.1.14) and AESIs leading to study early termination throughout the study period.

Any diagnosed episodes of COVID-19 will be reported as part of MAAEs; no active surveillance for SARS-CoV-2 infection is planned in this study.

Details on AE collection, follow-up and report are in Sections 9.1.7, 9.1.10 and 9.1.11.

Planned timepoints for all safety assessments are provided in the SoA.

8.10 Schedule of activities

The SoA is provided in Table 1; Screening (Day 1)

All screening assessments should be completed and reviewed so as to confirm that potential subjects meet all eligibility criteria. The investigator will keep a screening log to record the details of all screened subjects and to confirm the eligibility or record the reasons for screening failure, as applicable.

8.10.1 Pre-vaccination procedures (Day 1)

- Informed consent form (section 8.1).
- Demographics (section 8.2).
- Medical history, previous medication and vaccination and against Covid-19 (section 8.2).

  Physical exam (at the investigator’s discretion) and vital signs (systolic/diastolic blood pressure, respiratory rate, body temperature and heart rate) (Section 8.3).
- Pregnancy test (Section 8.4).

8.10.2 Vaccination and post-vaccination procedures (Day 1)

Conduct randomization (section 8.6) after confirming eligibility (section 8.5).

The following post-vaccination procedures will be performed on Day:

- Subjects will be observed for 30 minutes ± 10 minutes after vaccination in case of any adverse reaction.
- Careful subject training (or who will perform measurements) on how to measure local reactions (calipers) and body temperature (thermometer), how to fill out and how often to complete the daily card will occur at Visit 1.

Daily card instructions should include the following:

- The subject should understand that the timely daily card completion on a daily basis is a crucial component to take part in the study.
- The subject should be instructed on how to complete the daily card and how to make corrections in wrong entries.
- From the vaccination day, the subject will check approximately 6 hours after vaccination for specific types of reactions at the injection site (solicited local adverse reactions), any specific generalized symptoms (solicited systemic AEs), body temperature (preferably axillary), any other symptoms or change in subject's health status and any drugs given.
- If the subject feels unusual hot or cold during the day, they should check the body temperature several times and record the highest body temperature seen on that day in the daily card.
- Collection of body temperature, solicited local adverse reactions, solicited systemic AEs will continue for a total of 7 days in the daily card. Unsolicited AEs and medication collection in the daily card will continue for 28 days after vaccination (or until the night prior to the next clinical visit).

Daily cards will be the solely source document allowed for solicited local and systemic AEs (including body temperature measurements). The following additional rules apply to documenting safety information collected by the daily card and with a review at V2 with the physician to check data.

Note: Any solicited AE meeting any of the following criteria should be entered in the subjects' source document (see Appendix) and also as an AE in the AE CRF:

- Solicited local or systemic AEs leading to a visit to a healthcare professional (MAAE, Section 9.1.15).
- Solicited local or systemic AEs causing the subject to withdraw from the study or the subject is withdrawn from the study by the investigator (AE leading to withdrawal, see Section 7.1).
- Solicited local or systemic AE that would otherwise meet the definition of a SAE (Section 9.1.2).
- Solicited local or systemic AE continuing beyond 7 days after vaccination

8.10.3 Last visit procedures (Day 28)

On Day 28, subject blood should be drawn for serological immunogenicity test.

The investigator should complete the end of study eCRF page for all subjects given the study vaccine.

The subject should be informed on the vaccine received in the study.

9.0 ADVERSE EVENTS

The following sections provide definitions for AEs, procedures for collection, follow-up and communication to authorities.

AEs will be reported by subject (or, when appropriate, by a caregiver, surrogate or subject's legal representative) or identified in the study visit.

The investigator and any designees are in charge of detecting, documenting and recording events meeting the definition of an AE or SAE and remain in charge of following serious AEs, deemed related to the study vaccine or to the study procedures, or which led the subject to discontinue from the study (see Section 7.0).

9.1 Definitions

9.1.1 Definition of an adverse event

- An AE is any untoward medical occurrence in a subject or subject who took a drug, whether deemed or not as related to the study vaccine.
- NOTE: Therefore, an AE may be any unfavorable and unintentional (including an abnormal laboratory finding), symptom or disease (new or flared) transiently related to the use of a drug.

9.1.1.1 Events meeting the definition of an AE

- Any abnormal laboratory test results (hematology, clinical chemistry or urinalysis) or other safety assessments (e.g., electrocardiogram, radiological scans or vital sign measurements), including those worsening from baseline, deemed as clinically meaningful in the investigator's medical and scientific judgment.
- Flare of a chronic pre-existing or intermittent condition, including an increase in condition frequency and/or intensity.
- New conditions detected or diagnosed after study vaccine dosing, although they may be present prior to status start.
- Signs, symptoms or clinical sequelae of a suspected drug interaction.

9.1.1.2 Events NOT meeting the definition of an AE

- Any clinically significant abnormal laboratory findings or abnormal safety assessments, related to an underlying disease, unless deemed by the investigator as more serious than expected for the subject's condition.
- Medical or surgical procedure (e.g., endoscopy, appendectomy): The condition leading to the procedure is the AE.
- Cases when there was no unfavorable medical occurrence (social and/or convenience hospital admission).

- Foreseen daily fluctuations of disease(s) or pre-existing condition(s) present or detected at the study start without worsening.
- Pre-existing conditions or signs and/or symptoms present in a subject prior to the first study vaccination. These events will be recorded in the eCRF medical history section.

9.1.2 Definition of a SAE

A SAE is defined as any AE that, at any dose:

a) Results in death.

b) Is life-threatening.

The term "life-threatening" in the definition of "serious" refers to an event in which the subject was at risk of death at the time of the event. It does not refer to an event that, hypothetically, could have caused death if more serious.

c) Requires hospitalization or prolongs the existing hospitalization.

Overall, hospitalization means that the subject was retained (usually involving at least one overnight stay) in the emergency hospital or ward for observation and/or treatment that would have not been appropriate in the doctor's office or outpatient clinic. Complications occurring during hospitalization are AEs. If a complication prolongs hospitalization or meets any other serious criterion, the event is serious. In case of doubt whether the "hospitalization" occurred or was required, the AE should be deemed as serious.

Hospitalization for an elective treatment of a pre-existing condition without worsened from baseline is not deemed as an AE.

d) Results in deficiency/persisting disability.

- The term deficiency means a substantial disruption in someone's ability to perform normal life functions.
- This definition is not intended to include experiences of relatively minor medical significance, such as uncomplicated headache, nausea, vomiting, diarrhea, flu and accidental trauma (e.g., ankle sprain) that may interfere with or prevent daily life functions, but are not a substantial disruption.

e) Is a congenital anomaly/birth defect.

f) Is a medically important event.

- Medical or scientific judgment should be exercised to decide whether the SAE report is appropriate in other situations, such as clinically important events that may not be immediately life-threatening or result in death or hospitalization, but may put the subject at risk or may require medical or surgical intervention to prevent one of the results listed in the definition above. These events usually should be deemed as serious.
- Examples of said events include invasive or malignant cancer, intensive treatment at an emergency room or at home for allergic bronchospasm, blood dyscrasias or seizures not resulting in hospitalization or the development of drug dependence or abuse.

9.1.3 AEs and SAEs Recording

- When an AE occurs, the investigator is in charge of reviewing all event-related documents (e.g., hospital progress notes, laboratory reports and diagnostic reports).
- The investigator will then record all relevant information on the AE in the eCRF. Each event should be recorded separately.
- It is **not** acceptable for the investigator to send copies of subject's medical records to the sponsor's designee/sponsor instead of completing the relevant eCRF page.
- There may cases in which copies of medical records for some cases are requested by the sponsor representative/sponsor. In this case, all subject's identifiers, except from subject's number, will be written in chart copies prior to submitting to the sponsor/sponsor's designee.
- The investigator will try to establish a diagnosis for the event, based on signs, symptoms and/or other clinical information. Whenever possible, the diagnosis (not the individual signs/symptoms) will be documented as an AE.

9.1.4 Intensity assessment

The investigator will assess the intensity of each AE reported during the study and will assign it to one of the following categories, based on the Food and Drug Administration (FDA) Toxicity Rating Scale for Health Volunteer Adults and Adolescents Enrolled in Preventive Vaccine Clinical Trials:

- Mild (Grade 1).
- Moderate (Grade 2).
- Serious (Grade 3).
- Life-threatening (Grade 4).
- Fatal (Grade 5).

Note: An event is defined as "serious" when meeting at least 1 of the pre-defined results as described in the definition of a SAE, NOT when rated as serious. Serious is a category used to rate the intensity of an event; AEs and SAEs may be assessed as serious.

9.1.5 Causality assessment

- The investigator is obliged to assess the relationship between the study vaccine and the occurrence of each AE. The AE should be characterized according to the causality as:
  - A-Consistent Association:
  - A1-Product-related reaction: caused or triggered by the vaccine or by one or more of the vaccine components.
  - A2-Reaction related to the quality of vaccines: caused or precipitated by a change in the quality of a vaccine, including the diluents and materials (syringes and needles) used for its administration.

- A3- Immunization Error: caused by improper handling, prescriptions, and/or administration.
- A4-Anxiety Reaction related to immunization and/or vaccination stress-related response (ISRR)
- B-Undetermined Association:
- B1-Consistent temporal relationship, but no evidence in the literature to establish a causal relationship.
- B2-The investigation data are conflicting regarding causality.

C. Inconsistent or Coincident Association: not causally related to product, pre-existing conditions caused by factors other than vaccines.

D.- Unclassifiable: lack of adequate information for classification.

(Reference: Manual de Eventos Adversos Pós-Vacinação, Ministério da Saúde, 2021; WHO. Causality Assessment of an Adverse Event Following Immunization, 2018)

.

- The investigator will use medical judgment to determine the relationship.
- Alternative causes, such as underlying disease(s), concomitant therapy and other risk factors, as well as the temporal relationship between the event and study vaccine dosing, will be taken into consideration and investigated.
- The investigator will also see the product information for marketed products in their assessment.
- For every AE/SAE, the investigator should document in physician's notes that they reviewed the AE/SAE and provided a causality assessment.
- The Sponsor must receive a list of all AE/SAE.
- The investigator may change their opinion on causality based on follow-up information and send a SAE follow-up report with the updated causality assessment.
- Causality assessment is one of the criteria used to determine regulatory reporting requirements.

9.1.6 AE outcome assessment

The investigator will assess the outcome of all AEs recorded during the study as:

- Recovered/resolved.
- Recovering/resolving.
- Not recovered yet/not resolved yet.
- Recovered with sequelae/resolved with sequelae.
- Fatal (only SAEs).
- Unknown.

9.1.7 Time period and frequency to collect information on adverse and serious events

Medical occurrences starting prior to study vaccination, but after obtaining that informed consent, will be recorded in medical history/current medical conditions section of the eCRF, and not the AE section. Flare of a chronic pre-existing or intermittent condition, including an increase in condition frequency and/or intensity after the first vaccination, will be deemed as an AE.

For all subjects:

- Any AEs occurring within 30 minutes after each study vaccination should be reported in the eCRF.
- Any AEs determined by the investigator (or designee) as SAEs, AESIs (serious and not serious), MAAEs or AEs leading to early study or study vaccine termination (regardless the causal relationship) should be reported in the eCRF from the first vaccination to the completion of the last study-related subject procedure, which may include contact for safety follow-up (until the end of study).

All AESIs and SAEs will be recorded and reported to the sponsor or sponsor's designee immediately and under no circumstances it should exceed 24 hours after investigator's acknowledgment. The investigator will send any updated data to the sponsor within 24 hours after its availability.

9.1.8 Methods to detect AEs and/or SAEs

The method to record, assess the intensity, causality and outcome of AEs and/or SAEs and procedures to fill out and submit AESIs/SAE reports are provided in Section 9.1.7, Section 9.1.11 and Section 9.1.14.

care should be taken not to introduce bias when detecting AEs and/or SAEs. Open-ended oral questioning and non-leading to the subject is the preferred method to ask on AE occurrences.

9.1.9 Solicited and spontaneous adverse events

The investigator or designee will review and check the completed daily card during discussions with the subject, as per timepoints detailed in the SoA.

When applicable, subjects will record solicited AEs and presence of spontaneous AEs in the daily card. The investigator or designee will review daily card entries and will collect details of spontaneous AEs with the subject. The investigator or designee will then transcribe all proper information collected in relevant eCRF pages, as per guidance shown in Section 9.1.7. If the AE meets AESI or SAE criteria, it should also be reported to the sponsor or sponsor's designee (Section 9.1.11).

Solicited local reactions (injection site):

- Pain at the injection site.
- Erythema.
- Swelling.

Solicited systemic AEs:

- Fatigue.
- Headache.
- Myalgia.
- Arthralgia.
- Loss of appetite.
- Nausea.
- Chills.
- Fever (≥38.0 °C; axillary).

Seriousness rating for these AEs is shown in Table 7.

Table 7 Seriousness rating for solicited local reactions and systemic adverse events

	Mild (Grade 1)	Moderate (Grade 2)	Serious (Grade 3)	Life-threatening (Grade 4)
Pain	Not interfering with daily activities	Interfering with daily activities	Preventing daily activity	Required ER visit or hospitalization
Erythema	25-50 mm	51-100 mm	> 100 mm	
Swelling	25-50 mm	51-100 mm	> 100 mm	
Fatigue	Not interfering with daily activities	Interfering with daily activities	Preventing daily activity	Required ER visit or hospitalization
Headache	Not interfering with daily activities	Interfering with daily activities	Preventing daily activity	Required ER visit or hospitalization
Myalgia	Not interfering with daily activities	Interfering with daily activities	Preventing daily activity	Required ER visit or hospitalization
Arthralgia	Not interfering with daily activities	Interfering with daily activities	Preventing daily activity	Required ER visit or hospitalization
Loss of appetite	Eating less than normal/no effect on normal activity	Eating less than normal/interfering with normal activity	Not eating anything	Required ER visit or hospitalization
Nausea	Not interfering with daily activities	Interfering with daily activities	Preventing daily activity	Required ER visit or hospitalization
Chills	Not interfering with daily activities	Interfering with daily activities	Preventing daily activity	Required ER visit or hospitalization
Fever (°C)	38.0-38.4	38.5-38.9	>39.0	

Note: Based on 1: Guidance document from the Food and Drug Administration: Toxicity rating scale for healthy adult and adolescent volunteers enrolled in preventive vaccine clinical trials. ER, emergency room.

Unsolicited AEs

An unsolicited/spontaneous AE is an AE not listed as "solicited" above.

Potential spontaneous AEs may be seen by physicians (defined as symptoms or diseases requiring hospitalization or visit to the emergency room or visit to/by a healthcare professional; Section 9.1.15) or reason to concern for subjects. In case of said events, subjects will be instructed to contact the study site as soon as possible to report the event(s). Detailed information on spontaneous AEs reported will be collected by the qualified study site team during the interview and will be documented in the subject's records.

Spontaneous AEs not seen by physicians or noticed as a concern by subjects will be collected during planned safety visits/contacts with subjects and by review of medical records available.

9.1.10 AEs and SAEs Follow-up

- After AE/SAE report, the investigator should proactively follow each subject in subsequent visits/contacts. All AEs/SAEs will be followed to resolution, stabilization, event explained otherwise of subject lost to follow-up (as defined in Section 7.3). More information in follow-up procedures are provided in Section 9.1.10.
- The investigator is obliged to perform of arrange for the performance of supplementary measurements and/or assessments as indicated by the physician or as requested by the sponsor/sponsor's designee so as to elucidate the AE nature and/or causality as fast as possible This may include additional laboratory tests or investigations, histopathological tests or visits to other healthcare professionals.
- New or updated information will be recorded in the originally completed eCRF.
- The investigator will send any updated SAE data to the sponsor/sponsor's designee within 24 hours after receiving the information.
- A post-study AE/SAE is defined as any event occurring out of the AE/SAE reporting period. Investigators are not obliged to actively seek AEs or SAEs from former study subjects. However, in case the investigator becomes aware of any SAE, including death, at any time after a subject is discharged from the study, and they deem that the event is reasonably related to the study vaccine(s), the investigator will promptly notify the sponsor.

9.1.11 SAE regulatory report requirements

Immediate reporting of a SAE from the investigator to the sponsor is crucial so that legal and ethical responsibilities as for subject safety and study vaccine safety under medical investigation are met.

The sponsor is legally in charge of reporting to the local regulatory authority and other regulatory agencies on study vaccine safety under medical investigation. The sponsor will comply with country-specific regulatory requirements as for safety reports to the regulatory authority, Institutional Review Board (IRB)/IEC and investigators.

The investigator's safety reports should be prepared for suspected unexpected serious adverse reactions (SUSARs) as per local regulatory requirements and sponsor policies and sent to investigators as required.

An investigator receiving a safety report from the investigator describing a SAE or other specific safety information (e.g., SAE summary or list) from the sponsor will review it and then file it along with the IB and will inform the IRB/IEC, if appropriate as per local requirements.

An investigator who receives an investigator safety report describing an SAE or other specific safety information (e.g., summary or listing of SAEs) from the sponsor will review and then file it along with the IB and will notify the IRB/IEC, if appropriate according to local requirements.

9.1.12 AESIs and SAEs Reports

AESI and SAE reports may be done through electronic data capture (EDC; Section 9.1.12.1) or printed AESI/SAE form (Section 9.1.12.2).

9.1.12.1 AESI or SAE reports through eCRF page in the EDC

- The main mechanism to report an AESI or SAE to the sponsor/sponsor's designee will be the use of an electronic data collection tool (AE page in eCRF under EDC).
- If the electronic system becomes unavailable for over 24 hours, the study site will use the printed AEs/SAEs data collection tool (see next section) as a backup report type. The study site will enter AESI/SAE data back to the electronic system as soon as available.
- All initial and follow-up information should be reported within 24 hours after acknowledgment.

9.1.12.2 AESI and SAE report through printed AESI/SAE Form

- If the EDC is unavailable, AEs/SAEs may be reported to the sponsor/sponsor representative by using a printed AEs/SAEs form.
- All initial and follow-up information should be still reported within 24 hours after acknowledgment.
- Contacts (e-mail/fax) for printed AESI/SAE report may be found in AESI/SAE printed report form instructions.

9.1.13 Pregnancy

Females subjects at childbearing potential should have a urine pregnancy test [prior] to study vaccine dosing. The study vaccine may be given solely if the pregnancy test is negative.

Note: Pregnancy tests should be performed even if the subject is in her period at the study visit.

If a pregnancy is reported, the investigator should inform the sponsor within 24 hours after becoming aware of the pregnancy and they should follow the procedures described in Appendix 1.

9.1.14 Adverse events of special interest

Potential immune mediated diseases (pIMDs) include autoimmune and other inflammatory and/or disorders of interest that may or not have an autoimmune etiology. All AEs required to be recorded and reported as pIMDs included those listed in Appendix . However, the investigator will exercise their medical and scientific judgment when deciding whether other diseases have autoimmune origin (i.e., physiopathology involving systemic pathogenic or organ-specific autoantibodies) and should also be recorded as a pIMD.

The data safety monitoring board (DSMB) may adjudicate reported AEs meeting pIMD criteria.

To facilitate pIMD documenting in the eCRF, a standard pIMD questionnaire and a list of preferred terms (PTs) for the diagnoses above will be available to the investigator at the study start.

Once any AESI is diagnosed (serious or not serious), the investigator (or designee) should record the AESI in the relevant eCRF page and rapidly report the AESI, as per Section 9.1.11.

When there are evidences enough to make any of the diagnoses above, the AE should be reported as an AESI. Symptoms, signs or conditions that may (or not) represent the diagnoses above should be recorded and reported as AEs, but not as AESIs, until the final or definitive diagnosis has been determined and alternative diagnoses have been ruled out or shown to be less likely.

Furthermore, AEs potentially related to COVID-19 (Table 12) should be reported as AESIs. These events were defined by the Safety Platform for Emergency Vaccines (SPEAC Recommendations, 2020) and shown in Appendix 3 (Table 13).

9.1.15 Adverse events with medical care

MAAEs are defined as AEs with visits with medical care, including hospital, emergency room, urgent care clinic or other visits to medical personnel or thereof for any reason, but not meeting the seriousness criteria. Study routine visits are not deemed as medical care visits.

9.1.16 Assessment of Potential COVID-19 Cases

Study subjects will be asked on a COVID-19 history and any positive SARS-CoV-2 test results. In case a study subject develops suspected symptoms or signs of suspected COVID-19, a diagnostic investigation should be made. This assessment includes nasal, nasopharyngeal or oropharyngeal swab collection for SARS-CoV-2 test by RT-PCR and other diagnostic procedures as per the latest national guidance. The swab should be collected within 3 to 5 days after symptom onset. The test will not be repeated if the result is negative.

Any COVID-19 infection episodes diagnosed will be reported as part of MAAEs in the eCRF.

All SARS-CoV2 infection cases confirmed by RT-PCR will be rated as per the clinical progression scale proposed by the World Health Organization. The assessment of hospitalized cases (score 4 or higher) will be performed daily until symptom resolution. In non-hospitalized cases, the maximum score and symptom duration will be recorded (score 1-3).

In case of hospitalization due to COVID-19, all efforts will be made to collect information on the status seriousness in a proper study form.

9.2 Overdose treatment

Not applicable.

10.0 STATISTICAL CONSIDERATIONS

Study population: The entire population, excluding subjects with confirmed PCR for COVID-19.

Primary endpoint: Anti-Spike IgG in the study population 28 days after booster dose.

Secondary endpoint:

1. Neutralizing titer for SARS-CoV-2 Wuhan 28 days after the booster dose;
2. Neutralizing titer for SARS-CoV-2 variants (alpha, beta, gamma and delta) 28 days after the booster dose;
3. ELISA and other immunogenicity assays to check antibody persistence on Day 1;
4. Safety.

10.1 Statistical hypotheses

The study will use a non-inferiority design and the main research question is:

1. If anti-Spike IgG titers against wild type, induced in a heterologous booster dose schedule (3rd dose) (AstraZeneca/Fiocruz, Pfizer/Wyeth or Janssen) is non-inferior to that induced in a homologous booster dose schedule (3rd dose) (Sinovac/Butantan).

The study hypothesis is:

H_0: $GMC_{heterologous}/ GMC_{homologous} \leq 0.67$ or $\log_{10}(GMC_{heterologous}) - \log_{10}(GMC_{homologous}) \leq -0.174$;

H_1: $GMC_{heterologous}/ GMC_{homologous} > 0.67$ or $\log_{10}(GMC_{heterologous}) - \log_{10}(GMC_{homologous}) > -0.174$.

Where non-inferiority is shown, a second comparison analysis will be tested for superiority.

10.2 Sample Size Determination

The assumptions for sample size estimation are:

1. SD for neutralization titer is 0.5 in log10 scale and 0.4 for anti-Spike titer, based on current data received in ComCOV assays, 28 days after the second dose;
2. Non-inferiority margin of 0.67;
3. True GMR is 1;
4. Type I error = 0.0167;
5. Power = 90%

For the immunogenicity objective, the study will need to recruit at least 124 evaluable subjects per subgroup for the immunogenicity objective. Adjusting for intercurrent exclusion, PCR-positive, incomplete data and loss of follow-up will require approximately 1240 subjects in total accrued into 4 randomized screening groups with ratio (5:5:6:5).

The analysis set is defined in Table 8 .

Table 8 Analysis sets

Analysis set	Description
Enrolled set	All screened subjects who provided informed consent form and were given a subject ID, regardless subject randomization and treatment status.
Safety analysis set (SAS)	All subjects who were given a vaccine booster dose.
Modified intention-to-treat group (mITT)	All subjects who were randomized, received at least one dose of the study vaccines/comparators and provided post-vaccination immunogenicity data.
Per protocol set (PPS)	mITT subjects who were correctly given the full vaccination regimen provided post-vaccination immunogenicity data and no other major protocol deviation on Days 1 and 28.

10.3 Statistical Analysis

The Modified intention-to-treat group (mITT) population will be used for the primary objective of describing the immunogenicity of combined/booster regimens and includes all subjects who were given the study vaccine correctly and have available post-vaccination immunogenicity data.

A sensitivity analysis will be conducted using the per protocol analysis population.

The immunogenicity analysis, including GMT, GMFR will be produced for each group and subgroup for all timepoints available, along with 95% CIs.

GMT will be estimated as the anti-log of the mean of log-transformed values. Two-tailed 95% CI for GMT log will be obtained using the t distribution based on the results of log-transformed results, and then taking the anti-log of the CI to obtain the CI for GMT.

GMFR will limited to subjects without missing values at baseline and post-vaccination timepoint. GMFR will be estimated as the anti-log of the mean of the difference in log-transformed assay results (post-vaccine timepoint – baseline). Associated two-tailed CIs for

GMFR will be obtained by estimating the CIs using paired t distribution for the mean difference of log-transformed assay results and anti-log by back-transforming the confidence limits.

Missing data will not be imputed.

The Safety Set will be used for all safety analyses including all subjects who were given at least one study vaccine. For safety and reactogenicity analysis, descriptive summaries (mean, standard deviation, median, minimum, maximum, etc., for continuous variables, counts, percentages and 95% CI-related Clopper-Pearson for categorical variables) will be provided.

The final data base lock will take place when all subjects have completed the study.

The statistical analysis plan (SAP) will contain more details on the definition of analysis variables and analysis methodologies to address all study objectives.

10.3.1 Demographics and baseline characteristics analysis

Descriptive statistics (mean, standard deviation, median, minimum and maximum) for age, height, weight and body mass index, risk factor for serious COVID-19 at enrollment will be estimated overall and per study group.

Subjects disposition by sex, age, ethnic background (race, ethnicity) will be summarized overall and per study group.

10.3.2 Immunogenicity analyses

The methods for immunogenicity statistical analyses are described in Table 9. .

Table 9 Immunogenicity assessments

**Endpoint**	**Statistical analysis methods**
**Primary-**	Primary endpoints for immunogenicity will be assessed by GMT andGMFR for anti-Spike (S) IgG protein titers (by ELISA and other assays for antibody detection) on Days 1 and 28 for each vaccine booster regimen.<br>The following analyses will be performed:<br>• GMT and 95% CI I.<br>• GMFR and 95% CI.<br>And GMT ratio will be computed among groups on Days 1 and 28.<br>Missing data will not be imputed.

Abbreviations: CI, confidence interval; ELISA, enzyme-linked immunosorbent assay; GMFR, geometric mean fold increase; GMT, geometric mean of the titers

10.3.1 Safety analyses

The safety analysis will be assessed by reactogenicity summaries (solicited AEs) and spontaneous AEs/SAEs/MAAEs/AESIs per safety analysis group, which includes all subjects given at least one vaccine dose.

The methods for immunogenicity statistical analyses are described in Table 10.

Table 10 Safety analyses

Endpoint	Statistical analysis methods
Primary	• NA
Secondary	**Reactogenicity (solicited local and systemic AEs):**<br>• Reactogenicity will be assessed by frequencies and percentages of subjects who experienced each solicited AE along with 95% CIs for each symptom by any (seriousness), maximum seriousness (mild, moderate or serious, except for fever) for 7 days after each dose and after any dose per vaccine group.<br>**Spontaneous AEs, SAEs, MAAEs and AESIs:**<br>• Frequencies and percentages of subjects reported with at least one AE after 28 days of each vaccination will be summarized by vaccine group, along with 95% CIs by Clopper-Pearson method.<br>• Frequencies and percentages of subjects reported with at least one SAE, MAAE and AESI will be summarized from dose 1 throughout the study per vaccine group and 95% CIs by Clopper-Pearson method.<br><br>AEs will be coded by using the latest MedDRA version. AEs will be shown by SOC and PT per study group.<br>Clopper-Pearson method will be used to estimate CIs.<br>Data listings of all AEs will be provided per subject at the final analysis.<br>All safety analyses will be carried out in the Safety Set. Missing data will not be imputed.

Abbreviations: AE, adverse event; AESIs, adverse event of special interest; CI, confidence interval; MAAE, adverse event with medical care; MedDRA, Medical Dictionary for Regulatory Activities; PT, preferred term; SAE, serious adverse event; SOC, system organ class.

10.4 Analysis timepoint

For subjects, the analyses were planned as follows:

- Final analysis of all immunogenicity and safety data (until Day 28 in all groups).

11.0 REFERENCE

1. Li F. Structure, Function, and Evolution of Coronavirus Spike Proteins. *Annu Rev Virol.* 2016; 29:237-261
2. Zhou, P., X. L. Yang, X. G. Wang, B. Hu, L. Zhang, W. Zhang, H. R. Si, Y. Zhu, B. Li, C. L. Huang, H. D. Chen, J. Chen, Y. Luo, H. Guo, R. D. Jiang, M. Q. Liu, Y. Chen, X. R. Shen, X. Wang, X. S. Zheng, K. Zhao, Q. J. Chen, F. Deng, L. L. Liu, B. Yan, F. X. Zhan, Y. Y. Wang, G. F. Xiao, and Z. L. Shi. A pneumonia outbreak associated with a new coronavirus of probable bat origin. *Nature*, 2020; 579: 270-73.
3. Polack FP, Thomas SJ, Kitchin N, et al. . Safety and efficacy of the BNT162b2 mRNA covid-19 vaccine. *N Engl J Med*. 2020; 383:2603–2615.
4. Voysey M, Clemens SAC, Madhi SA, et al. Safety and efficacy of the ChAdOx1 nCoV-19 vaccine (AZD1222) against SARS-CoV-2: an interim analysis of four randomised controlled trials in Brazil, South Africa, and the UK. *The Lancet*. 2020; 397:99–111.
5. Jackson LA, Anderson EJ, Rouphael NG, et al. . An mRNA vaccine against SARS-CoV-2 - preliminary report. *N Engl J Med*. 2020; 383:1920–1931.
6. Davies NG, Jarvis CI; CMMID COVID-19 Working Group, Edmunds WJ, Jewell NP, Diaz-Ordaz K, Keogh RH. Increased mortality in community-tested cases of SARS-CoV-2 lineage B.1.1.7. *Nature*. 2021; 593:270-274.
7. Widge AT, Rouphael NG, Jackson LA, et al. . Durability of responses after SARS-CoV-2 mRNA-1273 vaccination. *N Engl J Med*. 2020; 384:80–82.
8. Pottegård A, Lund LC, Karlstad Ø, et al. Arterial events, venous thromboembolism, thrombocytopenia, and bleeding after vaccination with Oxford-AstraZeneca ChAdOx1-S in Denmark and Norway: population based cohort study. *BMJ* 2021; 373:n1114
9. Excler JL, Kim JH.. Novel prime-boost vaccine strategies against HIV-1. *Expert Rev Vaccines*. 2019; 18:765–779.
10. 17. Lu S. Heterologous prime-boost vaccination. *Curr Opin Immunol*. 2009; 21: 34651.]
11. Spencer A, McKay PF, Belij-Rammerstorfer S, Ulaszewska M, Bisset CD, Hu K et al. Heterologous vaccination regimens with self-amplifying RNA and Adenoviral COVID vaccines induce superior immune responses than single dose vaccine regimens in mice. *Nat Commun*. 2021;17:2893..
12. He Q, Mao Q, An C, Zhang J, Gao F, Bian L et al. Heterologous prime-boost: breaking the protective immune response bottleneck of COVID-19 vaccine candidates. *Emerg Microbes Infect*. 2021; 10:629-637.
13. Zaid A Alhinai MD , Nagi Elsidig MD , Countries with similar Covid-19 vaccination rates yet divergent outcomes: are all vaccines created equal?, International Journal of Infectious Diseases (2021), doi: https://doi.org/10.1016/j.ijid.2021.06.040.

12.0 APPENDIXES

Appendix 1. Collection of Pregnancy Information 58
Appendix 2 Regulatory, ethics considerations and study supervision 59
Appendix 3 Adverse events of special interest 64
Appendix 4 Investigator's Signature 68

Appendix 1. Collection of Pregnancy Information

Pregnancy Test:

- Women of childbearing potential should be enrolled solely after confirmation of menses and a negative highly sensitive urine pregnancy test.

Collection of pregnancy information

Females who become pregnant

The investigator will collect information on the pregnancy of any female subject who becomes pregnant during the participation in this study. The information will be recorded in the proper form and sent to the sponsor within 24 hours after becoming aware of subject's pregnancy

Reporting pregnancy information

Information will be recorded by the investigator in the proper printed pregnant report form and submitted to the sponsor/sponsor designee within 24 hours after becoming aware of a pregnancy case and knowing any pregnancy follow-up information

Although pregnancy itself is not deemed as an AE, any pregnancy complications (e.g., congenital anomalies/congenital defects and miscarriages) may also be reported as SAEs.

The outcome of all pregnancies (miscarriage, elective termination, ectopic pregnancy, vaginal birth or congenital anomaly) should be followed and reported regardless the fetal status (presence or absence of anomalies) or procedure indication, during the study

Appendix 2 Regulatory, ethics considerations and study supervision

Regulatory and ethics considerations

- This study will be performed as per protocol and with the following:
  - Ethics consensus principles obtained from international guidelines, including the Declaration of Helsinki and International Ethics Guidelines of the Council for International Organizations of Medical Sciences (CIOMS)
  - Applicable GCP ICH guidelines.
  - Applicable laws and rules.
- The protocol, protocol amendments, ICF and other relevant documents (e.g., advertisements) should be submitted to an Ethics Committee by the investigator and reviewed and approved by the Ethics Committee prior to study start.
- Any protocol changes will require the approval from the Ethics Committee and the regulatory authority, when applicable, before implementing changes made to the study design, except those changes needed to eliminate an immediate risk for the subjects.
- The investigator will be in charge of the following:
  - Provide written summaries of the study status to the Ethics Committee annually or with more often, as per the requirements, policies and procedures set forth by the Ethics Committee.
  - Inform the Ethics Committee on SAEs or other significant safety findings, as required by the Ethics Committee procedures.
  - Oversee the study performance at the study site and compliance of GCP requirements, ICH guidelines, Ethics Committee for clinical trials (if applicable) and all other applicable local rules.
- After reading the protocol, each investigator will sign the protocol signature page and will send a copy of the signed page to the sponsor or representative (Appendix 4) The study will not start at any study site where the investigator has not signed the protocol.

The sponsor will provide an insurance as per local guidelines and requirements, at least, for subjects from this study. The insurance forms will be kept in the study files.

Informed consent process

- The investigator or its representative will explain the study nature to the subject or their legally authorized representative and will answer all study-related questions.
- Subjects or their legally authorized representatives should be informed that their participation is voluntary. Subjects or their legally authorized representatives should sign an informed consent form as per local rules, ICH guidelines and the Ethics Committee or study site.

- Medical charts should include a statement that the written informed consent form was obtained before subject enrollment in the study and the date when the consent was obtained. The authorized person to obtain the informed consent should also sign the ICF.
- Subjects or their legally authorized representatives should have consented again to the most current ICF version(s) during their participation in the study.
- An original copy of the ICF(s) should be provided to the subject or their legally authorized representative.
- The investigator (or designee) should provide a "subject card" to each subject. In an emergency situation, this card serves to inform the treating physician that the subject is in a clinical trial and that relevant information may be obtained by contacting the investigator. The card should contain the main contact address and telephone number for information in the clinical trial, according to the Institution template. Subjects should be instructed to keep subject card with them at all times throughout the study duration.

A rescreened subject is not obliged to sign another ICF if the rescreen takes place within 14 days from the previous ICF signature date.

Data protection

- Subjects will be given a unique sponsor identifier. Any records of subjects or data sets transferred to the sponsor will contain solely the identifier; subject names or any information identifying them will not be transferred.
- The subject should be informed that their study-related personal data will be used by the sponsor as per the local data protection legislation. The level of disclosure should also be explained to the subject.
- The subject should be informed that their medical records may be examined by clinical quality assurance auditors or other authorized personnel appointed by the sponsor, by proper members of the Ethics Committee and regulatory authority inspectors.

Administrative Structure

The administrative structure for this study is shown in Table 11.

Table 11 Study Administrative Structure

Title	Organization in Charge
Study Operations Management	CRO
Medical Monitoring	CRO/study site
Trial Master File	CRO
Randomization Code	CRO
Data management	CRO
Medical supply management	Third party
Quality assurance audit	Sponsor
Biostatistics	CRO
Medical Writing	CRO
Laboratory Assessments	Third Parties and Sponsor

Abbreviations: CRO, Contract Research Organization

Clinical trial data disclosure

The study results should be reported within 1 year from the clinical trial study. Regardless the result, the sponsor will send to the regulatory authority data base a summary of clinical trial results within 1 year from the clinical trial end. It should be followed by a layman-friendly written summary.

Data Quality Assurance

- All study-related subject data will be recorded in eCRFs, unless transmitted to the sponsor or person electronically designated (e.g., laboratory data). The investigator is in charge of checking whether the data entries are accurate and correct, by electronically signing the eCRF.
- The investigator should keep an accurate documentation (source data) supporting the information entered in the eCRF.
- The investigator should allow study-related monitoring, audits, Ethics Committee review and inspections by regulatory bodies and provide direct access to source data documents.
- The sponsor or designee in charge of data management of this study, including source data quality check.
- Study monitors will perform ongoing source data check to confirm whether data entered in the eCRF by study site authorized personnel from source documents; that subjects' safety and rights are protected and that the study is being performed as per the currently approved protocol and any other study agreements, ICH GCP and all applicable regulatory requirements.

- Records and documents, including signed ICFs, related to this study performance should be retained by the investigator for 5 years after study completion as per Resolution 466/12, unless local rules or institutional policies require a longer retention period. No record may be destroyed during the retention period after written approval by the sponsor. No record may be transferred to another place or third party without informing the sponsor in writing.

Source documents

The investigator/institution should keep proper and accurate source documents and study records that include all applicable notes on each subject from the study site. Source data should also be relatable, legible, up to date, original, accurate and complete. Changes in source data should be traceable, should not redact the original entry and should be explained, if required (e.g., through and audit trail).

- Source documents provide evidences of the subject existence and proved the integrity of data collected. Source documents are filed in the investigator study sites.
- Data entered in the eCRF transcribed from source documents should be consistent with source documents or discrepancies should be explained. The investigator may need to request precision medical records or transfer records, depending on the study. Moreover, current medical records should be available.

Study Site and Study Termination

The sponsor designee reserves the right to terminate the study site or terminate the study at any time, for any reason, at sole sponsor's discretion. Study sites will be closed after study completion. A study site is deemed as closed when all required documents and study supplies have been collected and a study site closure visit has been performed. The investigator may start terminating the study site at any time, provided that there is a reasonable cause and enough notice is given in advance of the intended termination. The reasons for anticipated termination of a study site by the sponsor or investigator may include, among others:

- Investigator failure to comply with the protocol, IRB/IEC or local health authorities' requirements, sponsor procedures or GCP guidelines.
- Improper subject accrual by the investigator.
- Discontinuation of additional study vaccine development.

Publication Policy

Data generated by this study are confidential sponsor information. The sponsor will make study results publicly available. Publication policy as for the investigator and study site will be set forth in the Clinical Trial Agreement.

- The results from this study may be published or shown in scientific meetings. In case this is foreseen, the investigator agrees to send all manuscripts or abstracts to the sponsor prior to submission. This allows the sponsor to protect proprietary information and provide comments.
- The sponsor will comply with the requirements for study result publication. As per the editorial practice and standard ethics, the sponsor will usually support the publication of multicenter studies only in full and not as data from individual site data. In this case, a coordinating investigator will be mutually appointed.

Appendix 3 Adverse events of special interest

Table 12 List of adverse events of special interest for the study

**Neuroinflammatory disorders**	• Cranial nerve neuropathy, including paralysis and paresis (e.g., Bell's palsy).<br>• Optical neuritis.<br>• Multiple sclerosis.<br>• Transverse myelitis.<br>• Guillain-Barré syndrome, including Miller Fisher syndrome and other variants.<br>• Disseminated acute encephalomyelitis, including site-specific variants, e.g., non-infectious encephalitis, encephalomyelitis, myelitis, myeloradiculoneuritis.<br>• Myasthenia gravis, including Lambert-Eaton myasthenic syndrome.<br>• Demyelinating peripheral neuropathies, including:<br>- Chronic inflammatory demyelinating polyneuropathy.<br>- Multifocal motor neuropathy.<br>• Monoclonal gammopathy-related polyneuropathy.<br>• Narcolepsy.
**Musculoskeletal disorders**	• Systemic lupus erythematosus and related conditions.<br>• Systemic scleroderma (systemic sclerosis), including:<br>- Diffuse scleroderma.<br>- Calcinosis, Raynaud's phenomenon, esophageal dysmotility, sclerodactyly and telangiectasis syndrome (CREST).<br>• Idiopathic inflammatory myopathies, including:<br>- Dermatomyositis.<br>- Polymyositis.<br>• Anti-synthetase syndrome.<br>• Rheumatoid arthritis and related conditions, including:<br>- Juvenile idiopathic arthritis.<br>- Still's disease.<br>• Polymyalgia rheumatica.<br>• Spondyloarhtropathies, including:<br>- Ankylosing spondylitis.<br>- Reactive arthritis (Reiter's syndrome).<br>- Undifferentiated spondyloarthitis.<br>- Psoriatic arthritis.<br>- Enteropathic arthritis.<br>• Recurrent polychondritis.<br>• Mixed connective tissue disorder.<br>• Gout.
**Skin disorders**	• Psoriasis.<br>• Vitiligo.<br>• Erythema nodosum.<br>• Autoimmune bullous skin diseases (including pemphigus, pemphigoid and herpetiform dermatitis).<br>• Lichen planus.<br>• Sweet's syndrome.<br>• Localized scleroderma (morphea).

**Vasculitis**	• Great vessel vasculitis, including:<br>- Giant-cell arteritis (temporal arteritis).<br>- Takayasu arteritis.<br>• Medium and/or small vessel arteritis, including:<br>- Polyarteritis nodosum.<br>- Kawasaki's disease.<br>- Microscopic polyangiitis.<br>- Wegener granulomatosis (granulomatosis with polyangiitis).<br>- Churg-Strauss syndrome (allergic granulomatosis angiitis or eosinophilic granulomatosis with polyangiitis).<br>- Buerger's disease (thromboangeitis obliterans).<br>- Necrotizing vasculitis (skin or systemic).<br>- Vasculitis positive for anti-cytoplasm neutrophil antibody (unspecified type).<br>- Henoch-Schonlein purpura (immunoglobulin A vasculitis).<br>- Behçet's syndrome.<br>- Leukocytoclastic vasculitis.
**Blood disorders**	• Autoimmune hemolytic anemia.<br>• Autoimmune thrombocytopenia.<br>• Anti-phospholipid syndrome.<br>• Pernicious anemia.<br>• Autoimmune aplastic anemia.<br>• Autoimmune neutrophenia.<br>• Autoimmune pancytopenia.
**Liver disorders**	• Autoimmune hepatitis.<br>• Primary biliary cirrhosis.<br>• Primary sclerosing cholangitis.<br>• Autoimmune cholangitis.
**Gastrointestinal disorders**	• Inflammatory bowel disease, including:<br>- Crohn's disease.<br>- Ulcerative colitis.<br>- Microscopic colitis.<br>- Ulcerative proctitis.<br>• Celiac disease.<br>• Autoimmune pancreatitis.

**Endocrine disorders**	• Autoimmune thyroiditis (Hashimoto thyroiditis).<br>• Grave or Basedow's disease.<br>• Type 1 diabetes mellitus.<br>• Addison's disease.<br>• Polyglandular autoimmune syndrome.<br>• Autoimmune hypophysitis.
**Other**	• Autoimmune glomerulonephritis, including:<br>- Immunoglobulin A kidney disease.<br>- Rapidly-progressing glomerulonephritis.<br>- Membranous glomerulonephritis.<br>- Membranoproliferative glomerulonephritis.<br>- Mesangioproliferative glomerulonephritis.<br>- Tubulointerstitial nephritis syndrome and uveitis.<br>• Eye autoimmune diseases, including:<br>- Autoimmune uveitis.<br>- Autoimmune retinitis.<br>• Autoimmune myocarditis.<br>• Sarcoidosis.<br>• Stevens-Johnson's syndrome.<br>• Sjögren's syndrome.<br>• Alopecia areata.<br>• Idiopathic pulmonary fibrosis.<br>• Goodpasture's syndrome.<br>• Raynaud's phenomenon.

Table 13 List of adverse events of special interest relevant to COVID-19 (guidance document of the safety platform for emergency vaccine [SPEAC])

**AESI** **Rationale to include as an AESI (1, 2, 3, 4 and/or 5)**
AESIs included for being seen with COVID-19 [3, 4]
Acute respiratory distress syndrome
Multi-system inflammatory syndrome (children and adults)
Acute heart injury (includes myocarditis/pericarditis, heart failure, cardiomyopathy, coronary artery disease, arrhythmia))
Coagulation disorder (including thrombotic disorders, bleeding disorders)
Anosmia, ageusia
Injuries similar to athlete's foot
Erythema multiforme
Skin vasculitis in a single organ
Acute kidney injury
Acute liver injury
Acute pancreatitis NEW (December, 2020)
Rhabdomyolysis NEW (December, 2020)
Subacute thyroiditis subacute New (December, 2020)
**AESI included for having a proven or theoretical relationship with general immunization**
Anaphylaxis[1, 2]
Thrombocytopenia[1, 2, 3, 4]
Generalized seizure[1, 2]
Acute disseminated encephalomyelitis[4]
Guillain Barre syndrome[3, 4]
**AESI included for having a proven or theoretical relationship with specific vaccine platform(s)**
Peripheral nerve idiopathic facial paralysis Intranasal *E coli* vaccine adjuvant with heat-labile toxin
Vaccine-related enhanced disease 1 (measles inactivated by formalin/RSV; HIV), 2 (YF Dengue chimera), 5 (SARS/MERS-CoVs)

Abbreviations: AESI, adverse event of special interest. Notes: 1) Proven association with immunization covering several different vaccines. 2) Proven association with the vaccine that could be theoretically true for new vaccines for COVID-19. 3) Theoretical concern based on wild-type disease pathogenesis. 4) Theoretical concern related to viral replication during wild-type disease. 5) Theoretical concern for being shown in an animal model with ≥ 1 vaccine platform. * Acute kidney injury - international consensus definition proposed by the consensus specialist panel of Kidney Disease Improving Global Outcomes (www.kdigo.org): a) Increased blood creatinine by ≥0.3 mg/dl (≥26.5 µmol/l) within 48 hours; OR b) Increased blood creatinine for ≥1.5 times baseline, occurred or presumably occurred within the 7 days previous days OR c) Urine volume ≤0.5 ml/kg/hour for 6 hours.

Acute liver injury - definition used in most COVID-19 publications (but without international consensus):

Source:https://brightoncollaboration.us/wp-content/uploads/2021/01/COVID-19-updated-AESI-list.pdf. Accessed on March 29, 2021.

Appendix 4 Investigator's Signature

Protocol Title: A phase 4, randomized, controlled, single-blind study to assess the immunogenicity and safety of a third heterologous booster dose with recombinant covid-19 vaccine (AstraZeneca/Fiocruz), mRNA covid-19 vaccine (Pfizer/Wyeth) or recombinant covid-19 vaccine (Janssen) in previously vaccinated subjects against Covid-19 with two Sinovac/Butantan doses compared to a third dose of homologous booster dose of adsorbed inactivated covid-19 vaccine (Sinovac/Butantan) in adults.

PROTOCOL No.: RHH_001

VERSION: 3.0 – 13-Dec-2021

This protocol is a confidential sponsor communication. I confirm that I read this protocol, understood it and will work as per this protocol. I will also work consistently with the ethical principles originated in the Declaration of Helsinki and are consistent with the Good Clinical Practices and applicable laws and rules. The acceptance of this document comprises my agreement that no unpublished information herein will be published or disclosed without previous written approval from the sponsor.

Instructions for the investigator: SIGN and DATE this signature page. PRINT your name, title and study site name where the study will be performed. Return the signed copy to the CRO.

I read the full protocol and agree to carry out the study as per:

Investigator's Signature: ______________________________ Date: __________

Print name: ______________________________

Investigator title: ______________________________

Study site name/address: ____
